# Evolving fuzzy neural classifier that integrates uncertainty from human-expert feedback

**DOI:** 10.1007/s12530-022-09455-z

**Published:** 2022-08-15

**Authors:** Paulo Vitor de Campos Souza, Edwin Lughofer

**Affiliations:** Department of Knowledge-Based Mathematical Systems, Johannes Kepler Universitat Linz, Science Park 2 (6th Floor), Altenbergerstrasse 69, 4040 Linz, Austria

**Keywords:** Evolving fuzzy neural classifier, Class label uncertainty, User annotation feedback, Stream classification, Interpretability of fuzzy classification rules

## Abstract

Evolving fuzzy neural networks are models capable of solving complex problems in a wide variety of contexts. In general, the quality of the data evaluated by a model has a direct impact on the quality of the results. Some procedures can generate uncertainty during data collection, which can be identified by experts to choose more suitable forms of model training. This paper proposes the integration of expert input on labeling uncertainty into evolving fuzzy neural classifiers (EFNC) in an approach called *EFNC-U*. Uncertainty is considered in class label input provided by experts, who may not be entirely confident in their labeling or who may have limited experience with the application scenario for which the data is processed. Further, we aimed to create highly interpretable fuzzy classification rules to gain a better understanding of the process and thus to enable the user to elicit new knowledge from the model. To prove our technique, we performed binary pattern classification tests within two application scenarios, cyber invasion and fraud detection in auctions. By explicitly considering class label uncertainty in the update process of the EFNC-U, improved accuracy trend lines were achieved compared to fully (and blindly) updating the classifiers with uncertain data. Integration of (simulated) labeling uncertainty smaller than 20% led to similar accuracy trends as using the original streams (unaffected by uncertainty). This demonstrates the robustness of our approach up to this uncertainty level. Finally, interpretable rules were elicited for a particular application (auction fraud identification) with reduced (and thus readable) antecedent lengths and with certainty values in the consequent class labels. Additionally, an average expected uncertainty of the rules were elicited based on the uncertainty levels in those samples which formed the corresponding rules.

## Introduction

Evolving models are intelligent approaches capable of working with data online: They update their parameters dynamically and incrementally based on new incoming data, typically in form of a sequential data stream (Gama 2010), and can therefore tackle on-line learning problems (Kasabov 2007). All intelligent models have specific advantages and disadvantages. Combining two or more intelligent techniques allows leveraging their advantages to produce more accurate solutions to the problem at hand (de Campos Souza 2020).

Evolving fuzzy (neural) classifiers (EF(N)C) are able to extract granules in the form of rules that contain IF-THEN connections in their antecedent parts and embed linguistically readable terms in order to explain their consequents, which are represented by class labels. Thus, compared to other machine learning (ML) classifiers, they offer interpretability in the form of input feature interrelations for particular classes and may allow an advanced form of user interaction directly with the model. Pioneering work on how to establish and update such classifiers in an evolving context from streams was presented in Angelov et al. (2008), with several extensions proposed in Angelov and Zhou (2008), Lughofer and Buchtala (2013), Wang et al. (2013) or Angelov and Gu (2018) (the later for deep learning aspects); see Škrjanc et al. (2019) for a comprehensive survey. Accuracy and interpretability of evolving fuzzy neural classifiers depend heavily on the quality of the data they are fed to perform pattern classification tasks. In general, data collection processes should meet standardized scientific criteria to avoid distortions in the results produced (Angelov and Zhou 2008). However, some devices, procedures, or processes for data collection may be subjected to external influences that can generate uncertainty in the data (Hariri et al. 2019). Models that work, for instance, with robot controllers (Se et al. 2002), face recognition (Xu et al. 2014) and the internet of things in medical contexts (Al-Turjman et al. 2019) can be affected by uncertainty in the data collection. Generally, experts can identify distortions and assign a degree of uncertainty to individual samples collected (Solomatine and Shrestha 2009).

Data uncertainty has been thoroughly addressed in machine learning models (Ghahramani 2015), and in particular in evolving approaches in Leite et al. (2012, 2013), Pratama et al. (2016), Subramanian et al. (2014). The approaches in Leite et al. (2012, 2013) consider uncertainty in the rules’ consequents by providing an interval-based output value in addition to Takaki–Sugeno (TS)-like hyper-planes. In the approaches in Pratama et al. (2016) and Subramanian et al. (2014), a ‘higher level’ of uncertainty is addressed by type-2 fuzzy sets, which are able to represent the uncertainty in the definitions of the fuzzy sets themselves (and thus in the local granular parts these represent in the feature space). These approaches, however, address uncertainty in the form of measurement noise or natural variation of the (target) sample values. Furthermore, for the input feature values, uncertainty is addressed by evolving fuzzy (neural) classifiers in a straightforward way due to the automatic extraction of fuzzy sets (Hühn and Hüllermeier 2009). None of the evolving approaches published to date addresses uncertainty explicitly when provided by the user along the particular target (class) labels (e.g., as a real value in [0, 0.5], with 0 indicating no uncertainty in the class label, and 0.5 indicating full uncertainty)—they extract the parameters/structures in their models solely based on measurement variations and the noise content.

### Our approach

The main objective of this work was to develop an evolving fuzzy neural classifier that incorporates user feedback in the form of label uncertainty of the samples fed to the model by considering explicitly this uncertainty when updating and evolving the classifier. We integrated uncertainty levels within the range [0, 0.5] (i) in the update of the data cloud structure by extending the classical (unsupervised) SODA (Self-Organized Direction Aware) approach (Gu and Angelov 2018) to create a supervised SODA with uncertainty integration (termed as *SSODA-U*), based on which the fuzzy neurons are constructed by using n-uninorms (rather than classical t-norms as commonly used in ENFS approaches (Škrjanc et al. 2019)), and (ii) in the update of the consequent parameters using recursive least squares (RLS), which we extended to create an uncertainty-based RLS approach.

The first layer of the model features a fuzzification method capable of working with online samples and of updating the parameters of data clouds (i.e., their centers and standard deviation) which are formed based on the (local) data density. We also propose a supervised evaluation of the clouds’ identification with the model’s expected output in order to (a) find the best cloud to which a new sample should be assigned or (b) alternatively evolve a new cloud. Furthermore, class label uncertainty levels are used when updating the clouds and in the clouds’ evolution process. In the second layer of the model, the concept of n-uninorms allows the aggregation of all neurons in the first layer to build advanced IF-THEN fuzzy rules, not just allowing AND-connections between the antecedent parts (and features contained therein), but also OR-connections, which may greatly increase the flexibility in the interpretation of the classification rules. The rules also help to define the weights that connect the first two layers of the model (fuzzy inference system) with the neural aggregation network responsible for the model’s defuzzification process and, consequently, the model’s final outputs. These weights are learned incrementally by a modified RLS approach which integrates the uncertainty levels in the update.

The main highlights of our new approach are:

- Construction of a model that is capable of working online and updates its model hyper-parameters based on user/expert uncertainty information.
- Identification of the relevance of user input to the essence of the data and, consequently, the construction of a fuzzification process which is consistent with the data’s real behavior.
- Usage of an approach based on data density to build neurons from classification clusters, which is extended for considering label uncertainty in the cloud update and forming process.
- Extraction of advanced, interpretable rules that allow AND- and OR-connections with uncertainty information in the consequent labels and in the whole rule, depending on the uncertainty levels in the data used for updating the rules (clouds).
- An approach for decreasing the length of the rules (thus increasing their readability) by down-weighting input features which are less relevant to explain the stream classification problem.

The paper is composed as follows. Section 2 describes several state-of-the-art approaches for handling data uncertainty and user input, and how they work effectively in machine learning models. Section 3 presents the main theoretical concepts that underlie our model’s key components. Further, it describes the evolving fuzzy neural network architecture, the fuzzy neuron, and all aspects related to the incremental evolving training procedures of our evolving neuro-fuzzy classifier, and how uncertainty in user feedback is used for the construction of the model architecture and for model training. In Sect. 4, the results are compared to those of related SoA approaches, and the interpretation of the rules obtained is demonstrated. The results showed that considering class label uncertainty explicitly in the updating process of the EFNC could improve the accuracy trend lines, compared to fully (and blindly) updating the classifiers with uncertain labels in the data. Furthermore, we found that the integration of (simulated) label uncertainty below 20% still led to similar accuracy trends as using the original streams (unaffected by uncertainty). This demonstrates the robustness of our approach up to this uncertainty level. Finally, Sect. 5 concludes the paper.

## Literature review

### Data uncertainty

There are some external and internal uncertainties in the data collection process. These procedures can be influenced by human inference, computational procedures, or equipment used for data acquisition (Chatfield 1995) (e.g., due to measurement noise).

The process of measuring physical phenomena is a transfer of information from a source system to an operator via a measurement system. The interaction between a source system and a measurement system causes modification to both, which is coded in a vector for transferring the desired information. Consider, for example, the problem of measuring the temperature of a small body in a water reservoir by means of an encapsulated sensor element or a liquid inside glass. Even if the measurement system does not significantly change the source system, it must reach equilibrium with it, and the measurement may not have taken into account all relevant information phenomena. As a result, the sensor may indicate a reading that does not capture the desired phenomenon (Dong et al. 2009).

The physical quantity obtained in an experimental procedure is always an approximation of its real value. The theory of errors aims to determine the best possible value for the quantity and how much it can differ from the correct value. The best possible value is also called the best estimate or the experimental value of the measurand. Uncertainty can then be defined as an indication of how much the best value may differ from the actual value. In other words, uncertainty is an estimate of the error, that is, the error’s value if it could be measured (Chatfield 1995).

It is often the case in experiments that uncertainty is greater than the instrument’s error limit. This happens, for example, when the variable to be measured exhibits an intrinsically varying behavior. To determine the uncertainty of an experimental data set, some approaches can be used. Uncertainty and least squares regression have been addressed by the authors in Hodges and Moore (1972). The authors in Bi et al. (2005) modeled uncertainty for support vector models in classification problems. The work in Huijbregts et al. (2001) dealt with aspects of uncertainty in life cycle inventory problems, and the authors in Kirk and Stumpf (2009) assessed the impact of data uncertainty on Gaussian process regression models. To define the parameters of a problem, fuzzy neural networks use numerical data involving data collection. Since fuzzification processes transform data into fuzzy sets, a high degree of uncertainty in this data impacts the construction of the model architecture. In this scenario, it is essential to consider uncertainty in the construction of the model parameters and the fuzzy neurons (Schnute 1987). In fuzzy systems, fuzzy neural networks and fuzzy classifiers, this is typically achieved in a straightforward way Hühn and Hüllermeier (2009) during the formation, extraction or definition of the rule antecedent parts, which contain fuzzy sets (Zadeh 1965); these have a particular support for a part of the feature space (typically represented in the form of a linguistic term (Gacto et al. 2011)), and thus the wider the support becomes, the more uncertain the information contained in data set (Siler and Buckley 2005). Handling of uncertainty in the class labels has been loosely addressed in batch construction of fuzzy systems (see D’angelo et al. (2015) and Casalino et al. (2019)), and has, to the best of our knowledge, remained unaddressed in evolving fuzzy systems/classifier approaches.

### Clustering of data streams with uncertainty

Clustering plays an important role in our approach, as it provides the granulation of the input feature space into several clusters (each cluster (in the form of a cloud) is associated with a neuron, see subsequent section, and constructed using n-uninorms). The procedures applied use several ways of defining how the data will be grouped (clustered). In most approaches, the distance between samples is one of the most relevant criteria for defining the proximity to a cluster center (Zhang and Pal 2000; Pedrycz and Izakian 2014). Construction of fuzzy neural network architectures already uses fuzzy c-means Lemos et al. (2010), axiomatic fuzzy sets (Duan et al. 2018), approaches based on data density (Guimarães et al. 2020; Souza et al. 2020; Zhou et al. 2020; de Campos Souza et al. 2021a, b, c; de Campos Souza and Lughofer 2022b), polynomial nodes Zhang et al. (2020), Bayesian approaches (Souza et al. 2019b, 2021), and equally spaced membership functions (Souza 2018; Souza et al. 2019a, 2020).

In recent years, factors such as data uncertainty have been incorporated into the criteria for defining or updating data clusters. In this context, the factors that involve the uncertainty of samples can define the best allocation of a new sample within a cluster (Hamidzadeh and Ghadamyari 2020). The authors in (Hamidzadeh and Ghadamyari 2020) addressed several clustering techniques that consider data uncertainty, highlighting their techniques and roles and possible applications. Approaches such as CluStream (Aggarwal et al. 2003), DenStream (Cao et al. 2006), and D-Stream (Chen and Tu 2007) stand out, which have online and offline phases to work with stream data and to build clusters incrementally. Therefore, the incorporation of data uncertainty into the fuzzification process has been consistently addressed in the recent years (Hamidzadeh and Ghadamyari 2020). However, here we address uncertainty that is based on user annotation expertise or explicit user input and which is not calculated from the data—as handled by all the approaches mentioned above. Data uncertainty has recently been addressed by approaches described in Ge et al. (2020). However, unlike in our work, data uncertainties are evaluated based on random values in a time-delay model and not on user-related uncertainty during the collection or the evaluation of new information.

## An evolving fuzzy neural classifier architecture that considers uncertainty: structure and training approach

Our approach helps in particular the model’s architecture to define its logical structure (Kasabov 2001). The evolving model described in this paper supports most of the structure defined in de Campos Souza et al. (2019), mainly in the model’s architecture and the construction of Gaussian neurons. However, adjustments were made in the first layer (fuzzification) and in the model’s training process to include uncertainty levels in class labels and to achieve a more efficient update in the case of higher uncertainty. Unlike in our previous model, the first layer’s weights are not defined in an aleatory way. A process that increases interpretability of the fuzzy rules is incorporated to define the Gaussian weights based on the feature importance levels for the classification problem at hand. Another significant difference from the approach in de Campos Souza et al. (2019) is that no pruning criteria are applied to the fuzzy neurons in the second layer, but a control approach over the clusters formed by the fuzzification process, preventing irrelevant clouds from contributing to the construction of the Gaussian neurons.

**Fig. 1:**
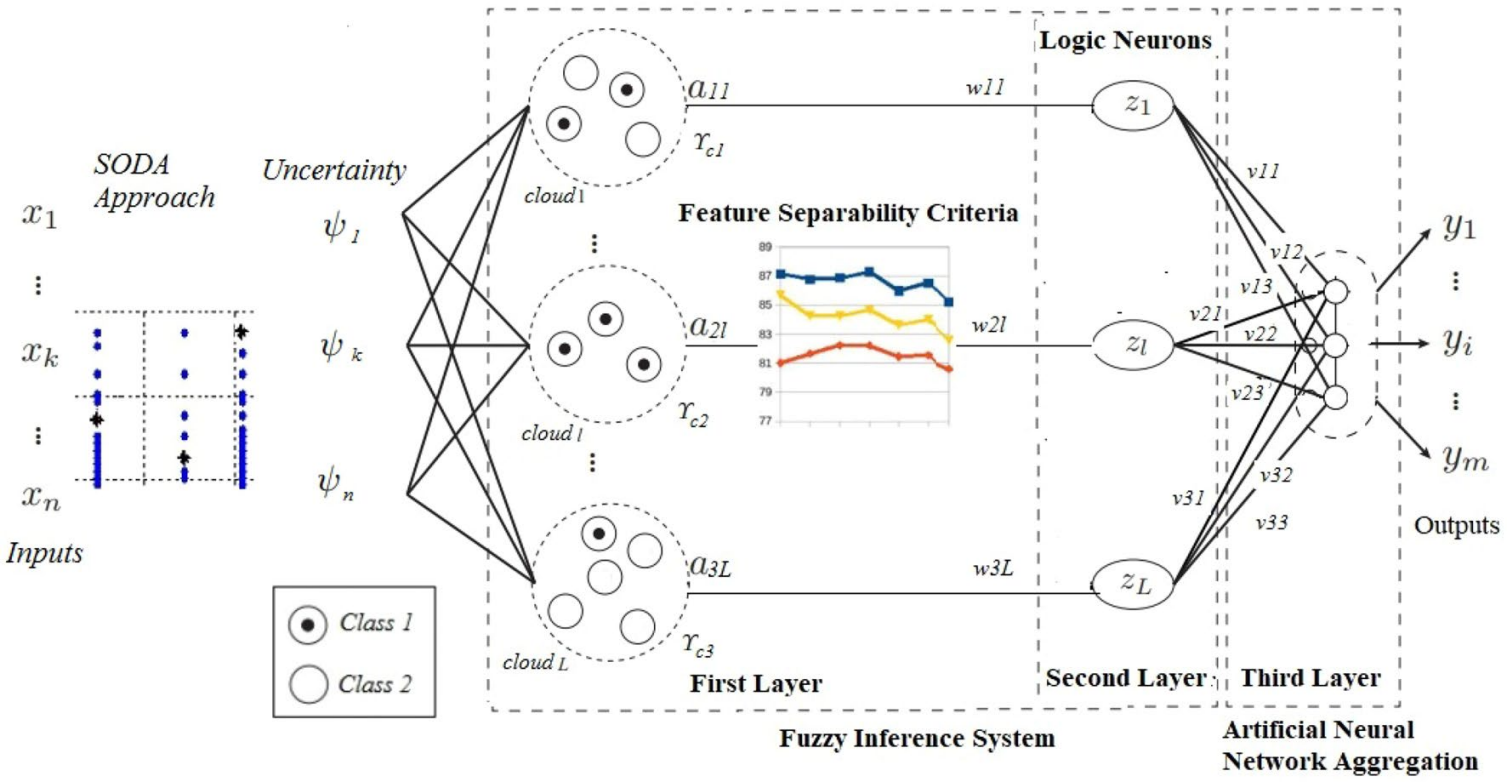
EFNC-U architecture

The model proposed in this paper has three layers in its architecture, where the first and the second are fuzzy inference based layers, and the third is a neural aggregation network layer responsible for defuzzification. Figure 1 illustrates the general feedforward topology of the evolving fuzzy neural classifier with uncertainty integration (EFNC-U) used in this paper. It uses the SSODA-U technique (as an extension of the classical SODA) to create neurons in the first layer for constructing the fuzzy neurons in the second layer. The first two layers form a fuzzy inference system which can create fuzzy rules that extract knowledge from the problem being analyzed.

### First layer

Essentially, the fuzzification process defines the first layer’s *L* neurons. The data clustering approach defines the centers (*c*) and sigmas ($\mathbf {\sigma }$) of the clusters that are the basis for forming the Gaussian neurons in the first layer. For each input feature $x_{j}$, *L* fuzzy sets are represented by $A_i^{j},j=1, \ldots , L$, whose membership functions are the activation functions of the corresponding neurons. Therefore, the final results of the fuzzification layer are the membership degrees associated with the input values:

1 $$\begin{aligned} a_{ij}=\mu (A^{j}_{i})\ for \ i=1, \ldots , \ N \ and \ j=1, \ldots , L \end{aligned}$$

where *N* is the number of inputs, and *L* represents the number of fuzzy sets (neurons) for each input (extracted from each cloud in each dimension). Therefore, in the case of Gaussian neurons formed in the first layer, the membership degrees can be expressed by:

2 $$\begin{aligned} a_{ij}(x_{i}, c_{ij}, \sigma _{ij}) = e^{-\frac{1}{2}\left( \frac{x_{i}-c_{ij}}{\sigma _{ij}}\right) ^2}, \ for \ \textit{i} = 1 \ldots \ \textit{N}, \ \textit{j} = 1 \ \ldots \ \textit{L} \end{aligned}$$

where $c_{ij}$ and $\sigma _{ij}$ are the center and width of the *j*th Gaussian function for input variable *i*, respectively.

After constructing the Gaussian neurons, a weight vector $w_{ij}$ (for $i = 1 \ldots N$ and $j = 1 \ldots L$)) is assigned to each of the *L* neurons in the first layer. They are usually defined at random in the range [0, 1]. However, we use an approach that extracts the weights based on the relationship between the input features and the classes in the stream classification problem. In particular, the weights are calculated to reflect how strong the discriminatory power of each feature among all classes is, based on a specified separability criterion. The feature weight proposal is based on a robust expansion of Fisher’s separability criterion (Dy and Brodley 2000), which was originally used in Lughofer (2011). Our approach uses the criterion in (3) for estimating the significance of each feature to discriminate between the classes (Dy and Brodley 2004):

3 $$\begin{aligned} J = trace(S_w^{-1}S_b) \end{aligned}$$

where *trace* defines the sum of the diagonal elements of the matrices $S_w$ and $S_b$. $S_b$ is the dispersion matrix between the classes, which measures the classes’ dispersion averages to the total average of the elements, and $S_w$ indicates the within-scatter matrix, which measures the samples’ dispersion in their class averages (Lughofer 2011). The class separability is directly related to the value of *J*: higher values of *J* indicate a better separability between the classes with the used feature set in $S_w^{-1}$ and $S_b$.

The approach presented in this paper uses each feature once, calculates $S_w^{-1}$ and $S_b$, and finally determines (3) for each feature $\rightarrow$ $J_1, \ldots ,J_N$, with *N* features in the data set. In this technique, the higher the value of $J_i$, the more important *i* is for discriminating among the classes. Thus, feature weights are then assigned by:

4 $$\begin{aligned} \mathbf {w_{j}} = \frac{J_j}{\max _{i=1, \ldots ,N} J_i} \end{aligned}$$

yielding always a weight 1 for the most important feature and a value in [0, 1] in relation to it for the other features. This approach defines the weights of the neuron in the first layer such that they correlate with the dimensions of the problem analyzed and down-weight less important features in the neuron construction. The incremental adaptation of $S_w^{-1}$ and $S_b$ with new incoming samples (which may change the feature importance for the classification problem), which induces an update of $J_i$ for each feature *i* by calculating (3), is described in detail in Lughofer (2011).

This type of procedure allows the neurons of the evolving fuzzy neural network to synchronize with the new data block’s relevance to the dimensions, making the learning process smooth in the fuzzy classifiers, as the evolution of the weights changes smoothly and continuously over time. This approach can be seen as a technique for selecting the essential features, since dimensions irrelevant to the problem will have values close to zero, which leads to dimensionality reduction in a soft manner (Lughofer 2011); soft because features with low weights close to 0 will have a marginal impact (and not no impact at all when they become exactly 0, as is the case in classical hard dimension reduction approaches) and can therefore be ignored when showing the final rules to the user (also see the results section for a concrete example). Thus, the rule length can be reduced and transparency improved.

Defining the weights of Gaussian neurons based on the problem’s features provides significant rules, since the impacts of rule antecedents will correlate with the resources they represent and will be directly linked to their ability to identify the problem’s classes. Hence, antecedents with an impact close to zero can be seen as irrelevant, which in turn creates more compact rules and thus improves their readability. Due to the continuous update of these weights, variations in the problem’s dimensions can be identified well; this means that features may become dynamically and autonomously reactivated due to new information in the data. This guarantees a smooth soft dimension reduction that does not require re-training phases with newly selected feature sets (as it would be the case when performing updates for hard feature selection/ranking approaches).

#### Supervised self-organizing direction-aware data partitioning with class label uncertainty—SSODA-U

The approach used in this paper for the first-layer fuzzification process builds on the self-organizing direction-aware data partitioning approach and extends it with the integration of data uncertainty (SODA-U). Thereby, the original (unsupervised) technique (SODA) proposed in Gu and Angelov (2018) was adapted and extended to create a supervised approach and to integrate class label uncertainty adequately. More specifically, our fuzzification algorithm is based on data density in order to integrate the samples’ class label uncertainty into the update of the clouds.

SODA is a data partitioning algorithm that builds on empirical data analysis (EDA) (Angelov et al. 2017b), which is commonly used to extract data clouds (clusters of arbitrary shape, which do not require any density function to be defined in advance) (Angelov and Yager 2012). These can then be used as a backbone for constructing fuzzy rules/neurons to achieve a suitable granulation of the input data space. SODA recognizes peaks/modes of the input frequency and applies them as focal points to join another feature to data clouds (similar to a Voronoi tessellation (Watson 1981)). The procedure for extracting data clouds is somewhat similar to clustering algorithms, with the main difference that they are non-parametric as they do not follow a pre-defined data distribution (Gu and Angelov 2018).

The method works with a magnitude component based on a universal distance metric and a directional/angular element based on the cosine similarity. The principal EDA operators were defined by the authors in Angelov et al. (2017b), suitable for processing data streams. We consider:

- ${R^{m}}$ = real data space,
- ($x_1, x_2,\ldots ,x_m$) = data stream sample,
- $x_i$ = [$x_{i1}, x_{i2},\ldots , x_{im}$] $^T \in {R^{m}}$ = m-dimensional vector where $m=1,2,3\ldots$ is the dimensionality of the problem and *i* represents the time instance at which the *i*th data sample is processed.

Consider the data set analyzed in the *n*th instance of time denoted by $x_1, x_2, \ldots , x_n$, then a sorted group of distinct variables from **x** is given by [$\epsilon _{i1}, \epsilon _{i2}, \ldots , \epsilon _{im}$]$^T$ $\in$ ${R^{m}}$. Still, on the perception of consciousness associated to the clustering proposed by Gu and Angelov (2018), consider as normalized numbers of the replications $f_1, f_2, \ldots , f_{n_{\epsilon }}$ where $n_\epsilon$ (greater than one and smaller than *n*) is the number of unique data samples.

The SODA approach uses the Euclidean distance metric to define the magnitude component (*M*), which can be represented for two samples $x_i$ and $x_j$ by Gu and Angelov (2018):

5 $$\begin{aligned} d_M(x_i,x_j)=\left\| x_i-x_j \right\| = \sqrt{\sum _{k=1}^{m} (x_{ik}- x_{jk})^2} i,j = 1,2\ldots ,n \end{aligned}$$

Also of relevance to this approach is the angular component. SODA implements the concept of cosine distance, which is defined as Gu and Angelov (2018):

6 $$\begin{aligned} d_A(x_i,x_j)= \sqrt{1- \cos {(\varTheta _{x_{i},x_{j}})}} i,j = 1,2\ldots ,n \end{aligned}$$

where $\cos {(\varTheta _{x_{i},x_{j}})} = \frac{<x_i, x_j>}{\left\| x_i \right\| , \left\| x_j \right\| }$ denotes the value of the angle between $x_i$ and $x_j$ and which is also known as cosine similarity Gu and Angelov (2018). Our fuzzification approach implements the measurement and angular component values mutually calculated between the samples on a 2D plane, called direction-aware (DA) plane Gu and Angelov (2018).

As previously mentioned, the empirical data analytics (EDA) operators (Angelov et al. 2017b) work with the cloud definition. The relevant operators used here are the cumulative proximity ($\pi$), the local density ($D_n$), and the global density ($D_n^G$):

7 $$\begin{aligned}&\begin{array}{c}\pi _n{\left( {\varvec{x}}_i\right) }=\sum \limits_{j=1}^nd^2{\left( {\varvec{x}}_i,{\varvec{x}}_j\right) }\end{array} \end{aligned}$$

8 $$\begin{aligned}&\begin{array}{c}D_n{\left( {\varvec{x}}_i\right) }=\frac{\sum _{j=1}^n\pi _n{\left( {\varvec{x}}_j\right) }}{2n\pi _n{\left( {\varvec{x}}_i\right) }}\end{array} \end{aligned}$$

9 $$\begin{aligned}&\begin{array}{c}D_n^G{\left( {\varvec{\epsilon }}_i\right) }=f_iD_n{\left( {\varvec{\epsilon }}_i\right) }\end{array} \end{aligned}$$

where $d(x_i, x_j)$ denotes the distance/dissimilarity between $x_i$ and $x_j$.

As can be seen from the preceding equations, they can be updated recursively. Using the Euclidean component (Eq. 5) allows recursive calculation of the cumulative proximity (Eq. 7). This can be expressed by Gu and Angelov (2018):

10 $$\begin{aligned} \begin{array}{c}\pi _n^M{\left( {\varvec{x}}_i\right) }=\sum\limits_{j=1}^nd_M^2{\left( {\varvec{x}}_i,{\varvec{x}}_j\right) }=n{\left( \left\| {\varvec{x}}_i-\varvec{\mu }_n^M\right\| ^2+X_n^M-\left\| -\varvec{\mu }_{\mathbf{n}}^{\mathbf{M}}\right\|^2\right) }\end{array} \end{aligned}$$

where $\varvec{\mu }_n^M$ and $X_n^M$ represent the mean of the data space and its squared norm mean, respectively. These two terms can be updated by Gu and Angelov (2018):

11 $$\begin{aligned}&\begin{array}{cc}\varvec{\mu }_n^M=\frac{n-1}{n}\varvec{\mu }_{n-1}^M+\frac{1}{n}{\varvec{x}}_n;\varvec{\mu }_1^M={\varvec{x}}_1\end{array} \end{aligned}$$

12 $$\begin{aligned}&\begin{array}{cc}X_n^M=\frac{n-1}{n}X_{n-1}^M+\frac{1}{n}\left\| {\varvec{x}}_n\right\| ^2;X_1^M=\left\| {\varvec{x}}_1\right\| ^2\end{array} \end{aligned}$$

The angular component updates similarly (Gu and Angelov 2018):

13 $$\begin{aligned} \begin{array}{cc}\varvec{\mu }_n^A=\frac{n-1}{n}\varvec{\mu }_{n-1}^A+\frac{1}{n}\frac{{\varvec{x}}_n} {\left\| {\varvec{x}}_n \right\|};&\varvec{\mu }_1^A=\frac{{\varvec{x}}_1}{\left\| {\varvec{x}}_1\right\| }\end{array} \end{aligned}$$

Using Euclidean distance to define the local density yields the following equation:

14 $$\begin{aligned} \begin{array}{c}\sum \limits _{j=1}^n\pi _n^M{\left( {x}_j\right) }=2n^2{\left( X_n^M-{\left\|-\varvec{\mu }_{\mathbf{n}}^{\mathbf{M}}\right\|}^2\right) }\end{array} \end{aligned}$$

This shows that the proposed SODA algorithm is able to process data streams in an incremental manner. In the SODA approach, understanding the concepts related to density is very helpful. This theme has been explored extensively by the authors in Angelov et al. (2017a, b). The local density $D_n$ is expressed as the inverse of the normalized cumulative proximity and directly indicates the main pattern of the data observed (Angelov et al. 2017a), where $D_n$ is defined as Gu and Angelov (2018):

15 $$\begin{aligned} D_n(x_i)=\frac{\sum _{j=1}^{n} \pi _n^M{\left( {x}_j\right) }}{2n\pi _n^M{\left( {x}_i\right) }} \end{aligned}$$

**Fig. 2:**
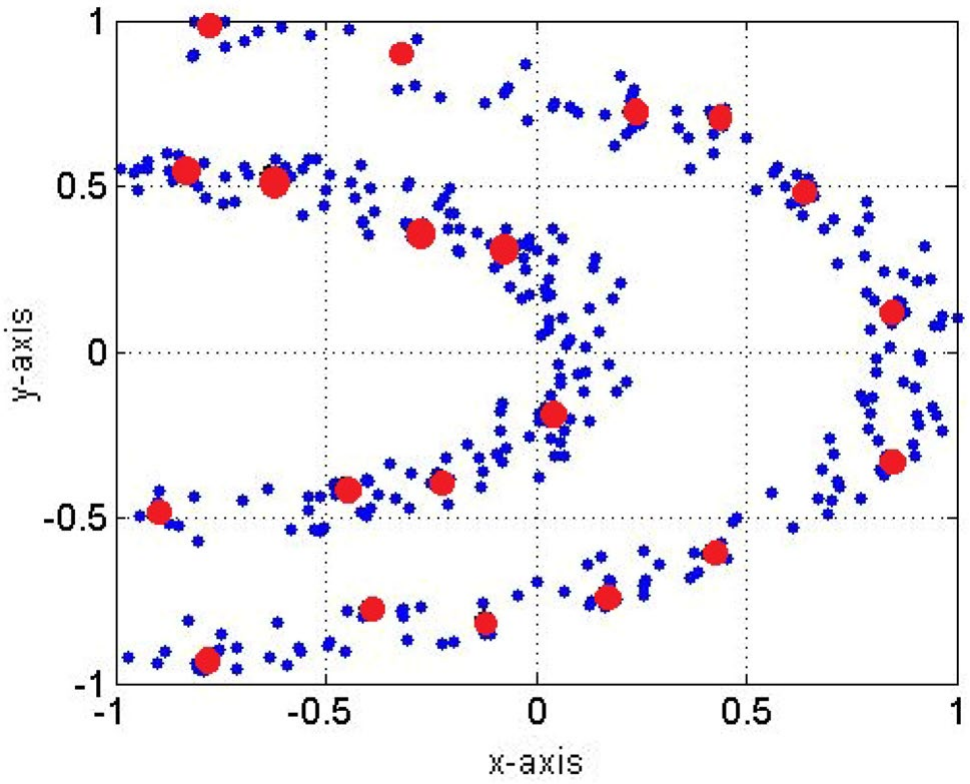
Cloud centers (large, red dots) according to the SODA algorithm

Figure 2 shows an example of the SODA definition and the centers (red points) of the density grouping defined by the algorithm.

The SODA algorithm’s key steps involve:

- first, form different DA planes of the recognized data samples, handling both the magnitude-based and angular-based densities;
- second, identify focal points;
- finally, use the focal points to partition the data space into data clouds Gu and Angelov (2018).

The algorithm comprises the following steps:

**Stage 1—Preparation**: Calculate the average values between every pair of data samples, $x_1, x_2, \ldots , x_n$ for both, the square Euclidean components $d_M$ and the square angular components, $d_A$.

16 $$\begin{aligned} {\overline{d}}_M^2= & {} \frac{\sum _{i=1}^n\sum _{j=1}^nd_M^2{\left( {\varvec{x}}_i,{\varvec{x}}_j\right) }}{n^2}\nonumber \\&=\frac{\sum _{i=1}^n\sum _{j=1}^n\left\| {\varvec{x}}_i-{\varvec{x}}_j\right\| ^2}{n^2}=2{\left( X_n^M-{\left\|-\varvec{\mu }_{\mathbf{n}}^{\mathbf{M}}\right\|}^2\right) } \end{aligned}$$

17 $$\begin{aligned} {\overline{d}}_A^2= & {} \frac{\sum _{i=1}^n\sum _{j=1}^nd_A^2{\left( {\varvec{x}}_i,{\varvec{x}}_j\right) }}{n^2}\nonumber \\&=\frac{\sum _{i=1}^n\sum _{j=1}^n\left\| \frac{{\varvec{x}}_i}{\mathbf {\left\| x_i\right\| }}-\frac{{\varvec{x}}_j}{\left\| {\varvec{x}}_j\right\| }\right\| ^2}{2n^2}=1-\left\| \varvec{\mu }_n^A\right\| ^2 \end{aligned}$$

After calculating the global density, the approach reclassifies the samples’ global density in a decreasing way, renaming them to $\{{\widehat{\varvec{\epsilon }}}_1,{\widehat{\varvec{\epsilon }}}_2,\ldots ,{\widehat{\varvec{\epsilon }}}_{n_u}\}$.

**Stage 2—DA plane projection:** The DA projection operation begins with the unique data sample with the highest global density, namely $u^-_1$. It is initially set to be the first reference, ${\mu }_1$ $\leftarrow$ $\widehat{\varvec{\epsilon }}_j$, which is also the origin of the first DA plane, denoted by $P_1$ (*L* $\leftarrow$ 1, *L* is the number of DA planes existing in the data space). The following rule can be expressed as the second step of the algorithm:

18 $$\begin{aligned} \begin{array}{c}\mathbf {Condition}\,\mathbf{1}\begin{array}{c}IF{\left( \frac{d_M{\left( {\varvec{\mu }}_l,{\widehat{\varvec{\epsilon }}}_j\right) }}{{\overline{d}}_M}<\frac{1}{\gamma }\right) }AND{\left( \frac{d_A{\left( {\varvec{\mu }}_l,{\widehat{\varvec{\epsilon }}}_j\right) }}{{\overline{d}}_A}<\frac{1}{\gamma }\right) }\\ THEN{\left( {{\mathbf{P}}_l\leftarrow {\widehat{\varvec{\epsilon }}}_j}\right) }\end{array}\end{array} \end{aligned}$$

where $\gamma$ assists on the decision of the granularity of the group used. Following the proposal in Gu and Angelov (2018), we used the value of $\gamma = 6$. When multiple DA planes meet Condition 1 at the same time, $\widehat{\varvec{\epsilon }}_j$ will be assigned to the closest one according to the following equation:

19 $$\begin{aligned} \begin{array}{c}i=\underset{l=1,2,\ldots ,L}{\arg \min }{\left( \frac{d_M{\left( {\varvec{\mu }}_l,{\widehat{\varvec{\epsilon }}}_j\right) }}{{\overline{d}}_M}+\frac{d_A{\left( {\varvec{\mu }}_l,{\widehat{\varvec{\epsilon }}}_j\right) }}{{\overline{d}}_A}\right) }\end{array} \end{aligned}$$

In this step, the mean, the support of the cloud, and the sum of the global density ($mu_i$, $S_i$ and $D_i$, respectively) are updated as follows:

20 $$\begin{aligned} \begin{array}{c}{\varvec{\mu }}_i\leftarrow \frac{S_i}{S_i+1}{\varvec{\mu }}_i+\frac{1}{S_i+1}{\widehat{\varvec{\epsilon }}}_j\end{array} \end{aligned}$$

21 $$\begin{aligned} \begin{array}{c}S_i\leftarrow S_i+1\end{array} \end{aligned}$$

22 $$\begin{aligned} \begin{array}{c}{\mathbf{D}}_i\leftarrow {\mathbf{D}}_i+D_n^G{\left( {\widehat{\varvec{\epsilon }}}_j\right) }\end{array} \end{aligned}$$

However, if Condition 1 is not met, a new DA plane ($P_{L+1}$) is created by setting its initial parameters as follows:

23 $$\begin{aligned} \begin{array}{c}{\varvec{\mu }}_L\leftarrow {\widehat{\varvec{\epsilon }}}_j\end{array} \end{aligned}$$

24 $$\begin{aligned} \begin{array}{c}S_L\leftarrow 1\end{array} \end{aligned}$$

25 $$\begin{aligned} \begin{array}{c}{\mathbf{D}}_L\leftarrow D_n^G{\left( {\widehat{\varvec{\epsilon }}}_j\right) }\end{array} \end{aligned}$$

Additionally, the number of overall clouds *L* is increased by 1:

26 $$\begin{aligned} \begin{array}{c}L\leftarrow L+1\end{array} \end{aligned}$$

This process is repeated until all samples of the initial data set have been visited. Some data samples may be located on several DA planes simultaneously, depending on the nature of the problem. In this case, the final affiliation of these samples is decided by the distances between them and the origin of the closest DA plane (Gu and Angelov 2018).

**Stage 3—Identifying the focal points**: For each DA plane, denoted as $P_e$, find the neighboring DA planes (${\{\mathbf{P}\}}_e^n, n=1,2,\ldots ,L, n\ne e$). The condition for deciding to evolve new focal points can be defined by the following relationship:

27 $$\begin{aligned} \begin{array}{c}\mathbf {Condition}\,\mathbf{2}\begin{array}{c}IF{\left( \frac{d_M{\left( {\varvec{\mu }}_e,{\varvec{\mu }}_l\right) }}{{\overline{d}}_M}\le \frac{2}{\gamma }\right) }AND{\left( \frac{d_A{\left( {\varvec{\mu }}_e,{\varvec{\mu }}_l\right) }}{{\overline{d}}_A}\le \frac{2}{\gamma }\right) }\\ THEN{\left( {{\left\{ \mathbf{P}\right\} }_e^n\leftarrow {\left\{ \mathbf{P}\right\} }_e^n\cup {\left\{ {\mathbf{P}}_l\right\} }}\right) }\end{array}\end{array} \end{aligned}$$

The main mode/peak of data density ($\mathbf {P_e}$) is determined if the following condition is met (where the corresponding **D** of ${\{\mathbf{P}\}}_e^n, n=1,2,\ldots ,L, n\ne e$ is expressed by ${\{\mathbf{D}\}}_e^n$ Gu and Angelov (2018)):

28 $$\begin{aligned} \begin{array}{c}\mathbf {Condition}\,\mathbf{3}IF{\left( {\mathbf{D}}_e > \max {\left( {\left\{ \mathbf{D}\right\} }_e^n\right) }\right) }\,THEN\,{\left( {\mathbf{P}}_e\,is\,a\,mode/peak\,of\,\mathbf{D}\right) }\end{array} \end{aligned}$$

All peaks have been found when Conditions 2 and 3 are met concurrently.

**Stage 4—Forming data clouds**: After all the DA planes for the modes/peaks of the data density have been identified, we define their origins, denoted by $\mu _o$, as the focal points for forming data clouds according to a Voronoi tessellation (Okabe et al. 2009). The expression that represents the data cloud is given by Gu and Angelov (2018):

29 $$\begin{aligned} \begin{array}{c}\mathbf {Condition}\,\mathbf{4}\begin{array}{c}IF{\left( v=\underset{j=1,2,\dots ,C}{\arg \min }{\left( \frac{d_M{\left( {\varvec{x}}_l,\varvec{\mu }_j^o\right) }}{{\overline{d}}_M}+\frac{d_A{\left( {\varvec{x}}_l,\varvec{\mu }_j^o\right) }}{{\overline{d}}_A}\right) }\right) }\\ THEN\,({\varvec{x}}_l\,is\,assigned\,to\,the\,v^{th}\,data\,cloud)\end{array}\end{array} \end{aligned}$$

where *C* is the number of focal points.

Note that the concept of data clouds is similar to the concept of clusters, except that they are non-parametric, do not have a specific shape, and can express any real data distribution that follows a concept of local density (Angelov and Yager 2012).

In our evolving approach, some steps are necessary for new samples to cause changes in the data clouds found. The following steps, which are connected to the steps listed previously, assist in defining the update of the SODA cluster method:

**Stage 5—Update parameters of existing clouds:** For each new incoming sample ${\varvec{x}}_{n}$, $\varvec{\mu }_n ^ M$, $\varvec{\mu }_n ^ A$ and $X_n^M$ are updated by (11)), (12)), and (13)). Similarly, the Euclidean components and the angular components between the new sample, and the centers of the DA planes are also updated for each new sample using Eqs. (5) and (6), and are then represented by $d_M{({\varvec{x}}_{n},{\varvec{\mu }}_l)}$ and $d_A{({\varvec{x}}_{n},{\varvec{\mu }}_l)}$, for $l=1,2,\ldots ,L$, respectively.

At this point in the process, the DA plane projection stage is triggered, allowing concurrent analysis of Condition 1 and Eq. (19) to determine whether the new sample belongs to an existing cluster (in this case, the model parameters are updated based on Eqs. (20) and (21)). If the new sample is unlike anything previously seen, a new DA plane is created using Eqs. (26), (23) and (24) Gu and Angelov (2018).

**Stage 6—Fusion of overlapping DA planes:** After the completion of Stage 5, the following condition is analyzed to identify strongly overlapping DA planes:

30 $$\begin{aligned} \begin{array}{c}\mathbf {Condition}\,\mathbf{5}\begin{array}{c}IF{\left( \frac{d_M{\left( {\varvec{\mu }}_i,{\varvec{\mu }}_j\right) }}{{\overline{d}}_M}<\frac{1}{2\gamma }\right) }\,AND\,{\left( \frac{d_A{\left( {\varvec{\mu }}_i,{\varvec{\mu }}_j\right) }}{{\overline{d}}_A}<\frac{1}{2\gamma }\right) }\\ THEN\,({\mathbf{P}}_i\,and\,{\mathbf{P}}_j\,are\,strongly\,overlapping)\end{array}\end{array} \end{aligned}$$

Since such overlaps are to be avoided, a new DA plane based on $P_j$ is created by merging the analyzed DA. This merger criterion is expressed by:

31 $$\begin{aligned} \begin{array}{c}L\leftarrow L-1\end{array} \end{aligned}$$

32 $$\begin{aligned} \begin{array}{c}{\varvec{\mu }}_j\leftarrow \frac{S_j}{S_j+S_i}{\varvec{\mu }}_j+\frac{S_i}{S_j+S_i}{\varvec{\mu }}_i\end{array} \end{aligned}$$

33 $$\begin{aligned} \begin{array}{c}S_j\leftarrow S_j+S_i\end{array} \end{aligned}$$

In the case of sample-wise updates from a stream, only the updated DA plane must be checked for significant overlaps with other DA planes. This process eliminates the parameters of the updated plane $P_i$ and then returns to Stage 5.

**Stage 7—Forming data clouds:** As soon as all samples have been analyzed, the SODA model identifies all the focal points of the existing centers in the DA planes that resulted from Stage 6. The following steps are linked to the global density calculation of the centers of the DA plane by Eq. (9), using the support of each DA plane as the number of iterations to be performed. This yields the global density, $D_n^G{({\varvec{\mu }}_l)}$. Each DA plane is then analyzed using Condition 2 to find its neighboring DA plan. After this, Condition 3 is used in order to verify whether $D_n ^ G {({\varvec{\mu }} _e)}$ is one of the local maxima of ($D_n ^ G {({ \varvec{\mu }} _l)}$). Finally, all maximum $D_n ^ G {({ \varvec{\mu }} _l)}$ locations identified and the centers of their corresponding DA planes are used to construct the focal points and consequently the data clouds.

At the beginning of the modelling stage, the SODA algorithm’s behavior will resemble offline behavior (Stages 1–4). When new data is subsequently fed to the model, SODA acts in its evolving form, with a primary focus on the evolving stages (Stages 5–7).

**Fig. 3:**
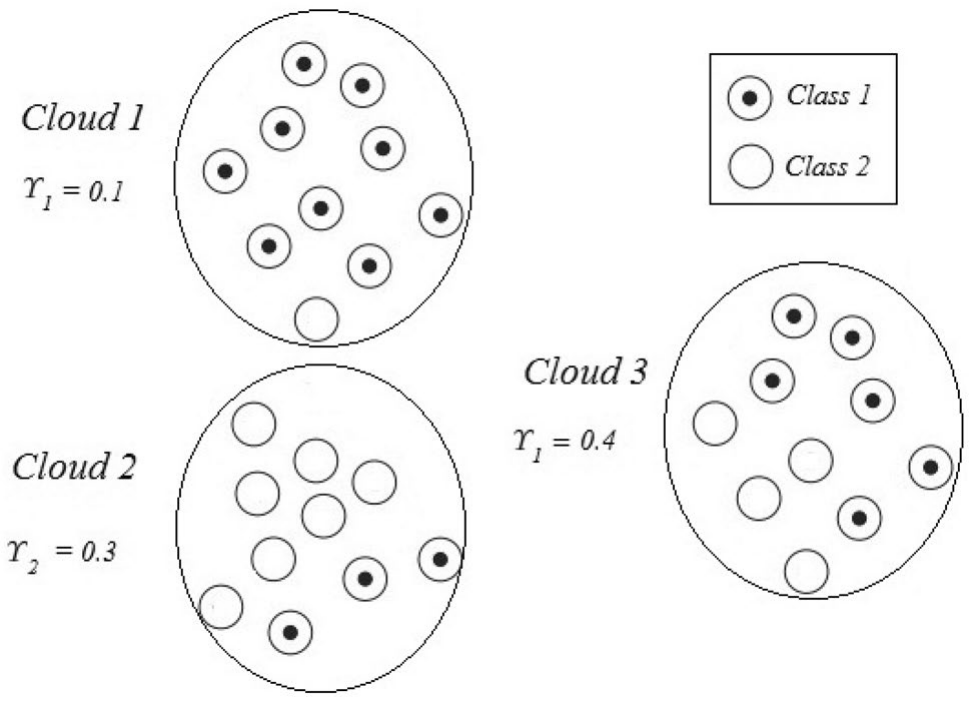
Uncertainty of clusters in relation to their majority class

The integration of label uncertainty is now done in the following way. As training is supervised, at the end of the SODA offline stage the number of samples that belong to each cloud can be checked. It is therefore possible to verify the uncertainty of a cloud in representing a particular class. This cloud uncertainty $\Upsilon _c$ for the majority class *c*, that is, the class which has the most samples over all classes within a cloud, is given by the ratio between class *c* samples and the total samples belonging to that cloud. Thus, for each class, a cloud that represents it better can be identified (that with the highest uncertainty in the respective class). This cluster uncertainty in the majority class can be calculated by:

34 $$\begin{aligned} \Upsilon _c= 1- \frac{S_{cL}}{S_L} \end{aligned}$$

where $S_{cL}$ and $S_L$ represent the number of samples belonging to the majority class and the total number of samples assigned to that cloud, respectively. Figure 3 illustrates uncertainty in clouds, showing three clouds with different majority classes. The uncertainty about the main class clouds is expressed by the number of samples that belong to Class 1 (first and third cloud) or Class 2 (second cloud) relative to the total number of samples belonging to each cloud.

In this work, we chose the class label uncertainty $\psi _i$ in the *i*th sample as being (actively) informed by the user (e.g., she/he is not entirely confident in her/his annotation to a degree of $\psi _i$) or based on the user’s experience with the process under study (typically estimated/defined in advance). Class label uncertainty is used to evaluate cluster allocation in online training (when the majority class *c* has the same label as the sample analyzed) under the following condition:

35 $$\begin{aligned} \begin{array}{c}\mathbf {Condition}\,\mathbf{6}\begin{array}{c}IF{\left( \psi _i<\Upsilon _c\right) }\\ THEN(Start \ Stage \ 7)\end{array}\end{array} \end{aligned}$$

When the majority class of the cloud differs from the sample label, another closer cloud is sought to verify the Condition 6 in . If there is no closer cloud with majority class equal to the sample’s class, the new sample forms a new cloud.

If the sample’s degree of uncertainty is higher than the degree of uncertainty of the cloud to which it was assigned, another cloud in proximity is sought that has a lower degree of uncertainty than the sample (related to the same majority class). If in the new assessment of cloud and sample uncertainty Equation (35) is satisfied, Eqs. (12), (13) and (14) are applied again. If neither cloud fulfills this condition, the sample initiates a new cloud of which it becomes the center. To prevent the number of clouds from growing uncontrollably and thus impacting the fuzzy rules, an abnormal-cloud-exclusion approach was applied to detach new clouds from the model and control their complexity. This guarantees that a sample with a very high degree of uncertainty is excluded from the fuzzification process and does not interfere with the update of the data density, the global cloud density, and other essential elements.

The idea behind this approach is that, if a new sample is analyzed and its uncertainty is higher than that of the cloud it was initially assigned to, it is not assigned to in that cloud and is destined for another, neighboring cloud. For example, consider that a new sample submitted to the model has $\psi$= 0.15 and that it was initially attributed to Cloud 1 (Fig. 3). As its degree of uncertainty is high compared to that of the cloud (0.15 > 0.1), the sample will not be assigned to this cloud but to the closest one with the same majority class for which it was originally intended (Cloud 3 in this example). Assessing the uncertainties of Cloud 3 and of the sample using Eq. (35), shows that the sample should be assigned to this cloud. If the sample’s $\psi$ were 0.45, it would not meet the criteria established in Eq. (35) for the two clouds to which it could be assigned. In this case, this sample would trigger the evolution of a new cloud, but the exclusion criterion for abnormal clouds would be applied, which would exclude this sample (with a high degree of uncertainty) from the model’s fuzzification process.

### Second layer

The layer responsible for building fuzzy rules is composed by *L* fuzzy logic neurons extracted by the SSODA-U approach (as one cloud corresponds to one neuron). Each fuzzy logic neuron performs a weighted aggregation of all first-layer outputs. This aggregation is performed using the feature weights $w_{ij}$ (for $i = 1\ldots N$ and $j = 1\ldots L$) as calculated by (4). For each input variable *j*, only one first-layer output $a_{ij}$ is defined as input to the *j*th neuron. These neurons can extract knowledge from the data set and generate fuzzy rules. This type of neuron can be seen as a fuzzy logic equation, which allows extraction of knowledge in the form of IF-THEN rules (Pedrycz and Gomide 1998). In our approach, we use n-uninorms which allow the connections between rule antecedents to be represented by ANDs or ORs, offering greater interpretability than classical t-norms (which use only AND-connections).

The neurons in this layer use fuzzy operators to aggregate the first-layer elements. To this end, the t-norm (*t* or *T*) was used as a product and the t-conorm (*s* or *S*) as a probabilistic sum. The most traditional fuzzy neurons are the and-neuron and the or-neuron, which are constructed by:

36 $$\begin{aligned} {\textbf {z}}= & {} AND ({\textbf {w;\,a}})=T^N_{i=1} (w_i \ s \ a_i) \end{aligned}$$

37 $$\begin{aligned} {\textbf {z}}= & {} OR ({\textbf {w;\,a}})=S^N_{i=1} (w_i \ t \ a_i) \end{aligned}$$

where **a** = $[a_1, a_2, \ldots , a_3, \ldots a_N]$ represents the Gaussian neurons in the first layer and **w** = $[w_1, w_2, \ldots , w_3, \ldots w_N]$ are their respective weights, with *N* the number of inputs, thus **a, w** $\in$ $[0,1]^N$.

Although they are useful for building fuzzy rules, they generate rules with the same antecedent connector (when using the and-neuron, only AND type connectors are generated in the rules). To create heterogenic fuzzy rule bases, that is, AND-rules and OR-rules, specific neurons termed unineuron (Lemos et al. 2010) and nullneuron (Hell et al. 2008) are employed. To aggregate neurons in the first layer of the model they use fuzzy operators, called uninorm (Yager and Rybalov 1996) and nullnorm (Calvo et al. 2001). These two types of fuzzy operators allow flexibility in creating fuzzy rules, as both can act as and-neuron or or-neuron, depending on the value of the identity element (uninorn) or the absorption element (nullnorm), which are both set in ]0,1[. Despite having a similar structure, these two operators have different functions regarding the variation of identity and absorption elements. The closer to zero the identity element, the more the fuzzy operator resembles a t-norm to operate its functions ($\rightarrow$ AND); the closer to 1, the more similar the operator becomes to a t-conorm ($\rightarrow$ OR).

Studies have shown that these operators satisfy commutativity, monotonicity, associativity, and identity (Yager and Rybalov 1996). In this paper, the uninorm and nullnorm operators are represented by:

38 $$\begin{aligned} U(x,y, g)= & {} \left\{ \begin{array}{ll}g \ T(\frac{x}{o},\frac{y}{g}), & if \ \textit{y} \ \in [0,g] \\ g + (1 - g)\ S \ (\frac{x-g}{1-g},\frac{y-g}{1-g}), & if \ \textit{y} \ \in \ (g,1] \end{array}\right. \end{aligned}$$

39 $$\begin{aligned} NU(x,y,u)= & {} \left\{ \begin{array}{ll}uS(\frac{x}{u},\frac{y}{u}), & if \, \textit{y} \ \in [0,u] \\ u \ + \ (1 - u)\ T\ (\frac{x-u}{y-u},\frac{1-y}{1-u}), & if \ \textit{y} \ \in \ (u,1] \end{array}\right. \end{aligned}$$

Unineurons and nullneurons use first-layer input processing and their respective weights at two levels. At the first level, the input signals submitted to the structure are calculated individually using the weights. At the second level, the results of all first-level components are aggregated and transformed into unique values (p-function). These fuzzy operators use common processes in which each pair ($a_i$, $w_i$) is converted into a value $b_i$ = **h** ($a_i$, $w_i$). At the second level, the aggregation of all previous values is calculated using a logic operator $\zeta$ ($b_1, b_2 \ldots b_N$), where $\zeta$ can be represented by unineurons (*U*) or nullneurons (*NU*). This operation can be represented mathematically by:

40 $$\begin{aligned} p(w,a,\chi )= wa+\bar{w}\chi \end{aligned}$$

where $\chi$ is the identity or the absorption element, depending on the neuron used in the model, and $\bar{w}$ represents the complement of *w*.

The uni-nullneurons can be represented generically by:

41 $$\begin{aligned} z=N_{un-null} (w;a;\chi )=\zeta ^N_{i=1} p(w_i, a_i, \chi ) \end{aligned}$$

These neurons facilitate the construction of more flexible fuzzy rules.

To leverage the dynamics of using these two operators together, the author in Akella (2007) proposed operators called n-uninorms, which generalize uninorm and nullnorm within the same fuzzy operator. Several n-uninorms meet these operators’ specifics, (see Akella (2007)). However, here we use an operator called 2-uninorm, which allows the use of uninorms and nullnorms in the same operator.

Akella defined a function *V*: $[0, 1]^{2}$ $\rightarrow [0,1]$ as a commutative binary function with $g, u, \beta \in$ [0,1] with $0 \le g \le \beta \le u \le 1$ and $0< \beta <1$. Therefore, $\{g, u\}_\beta$ is the definition of a 2-neutral element of *V* if it satisfies Akella (2007):

- V(*g*, x) =x for all x $$\le \beta$$
- V(*u*, x) =x for all x $$\ge \beta$$

The 2-neutral element defines the binary operator called 2-uninorm, $N^{un}$ = $[0, 1]^{2}$ $\rightarrow [0,1]$, if the operator is commutative, associative and has $\{g, u\}_\beta$ Zhou and Liu (2020). The $N^{un}$ operator can act as a uninorm if $g = \beta = u$, and can turn into a nullnorm if $g = 0$ and $u =0$. This behavior of the 2-uninorm operator is flexible regarding its operators and, as $0< g< \beta< u < 1$, it can be written as follows Zhou and Liu (2020):

42 $$\begin{aligned} N^{un}(x,y,\beta , g, u)=\left\{ \begin{array}{ll}\beta U_1(\frac{x}{\beta },\frac{y}{\beta }),& if \ \textit{x},\textit{y} \ \in [0,\beta ]\\ \beta \ + \ (1 - \beta )\ U_2\ (\frac{x-\beta }{1-\beta },\frac{y-\beta }{1-\beta }), & if \ \textit{x},\textit{y} \ \in \ (\beta ,1] \end{array}\right. \end{aligned}$$

where $U_1$ is a uninorm (Eq. (38,39)) with a neural element (identity) = $\frac{g}{\beta }$ and $U_2$ is a uninorm (Eq. (38,39)) using ($\frac{u-\beta }{1-\beta }$) as a neutral element. The properties of this operator have been described in Zhou and Liu (2020). In general, it is possible to verify that this operator uses the identity and absorption elements and a third element to define which operations will be performed by the operator.

The uninull-neuron is a fuzzy neuron that uses a 2-uninorm as a fuzzy operator to build fuzzy rules. This neuron also has two-level processing, with the Gaussian neurons’ inputs and their respective weights in the first layer. Therefore, based on Eq. (40), we can define the p-function as de Campos Souza and Lughofer (2022a):

43 $$\begin{aligned} p(w,a,\beta , g, u)=\left\{ \begin{matrix} wa+\bar{w}\frac{g}{\beta },\ if \ U_1 \\ wa+\bar{w}\frac{u-\beta }{1-\beta }, \ if \ U_2 \end{matrix}\right. \end{aligned}$$

Thus Eq. (41) can be adapted to represent the uninull-neuron as de Campos Souza and Lughofer (2022a):

44 $$\begin{aligned} z=UNI^{NUL} (w,a,\beta , g, u)=N^{un_{i=1}^N} p(w_i, a_i, \beta , g, u) \end{aligned}$$

The fuzzy rules generated by uninull-neurons can be expressed by:

45 $$\begin{aligned} \begin{aligned} Rule_1: \ If x_{i1} \ is \ A_1^1 \ with \ impact \ w_{11}\ldots \\ AND/OR_{(g,u,\beta )} \ x_{i2} \ is \ A_1^2 \ with \ impact \ w_{21} \ldots \\ AND/OR_{(g,u,\beta )} \ x_{iN} \ is \ A_1^N \ with \ impact \ w_{N1} \ldots \\ Then \ y_1 \ is \ v_1\\ with \ average \ uncertainty \ \varPsi _1\\ \ldots \ldots .. \\ Rule_L: \ If x_{i1} \ is \ A_L^1 \ with \ impact \ w_{1L} \ldots \\ AND/OR_{(g,u,\beta )} \ x_{i2} \ is \ A_L^2 \ with \ impact \ w_{2L} \ldots \\ AND/OR_{(g,u,\beta )} \ x_{i2} \ is \ A_L^N \ with \ impact \ w_{NL} \ldots \\ Then \ y_L \ is \ v_L \\ with \ average \ uncertainty \ \varPsi _L.\\ \end{aligned} \end{aligned}$$

The average uncertainty per rule $\varPsi _i$ can thus be calculated by the average uncertainty in the class labels of the samples which formed the rule, that is, for which their cloud representation was the closest, and thus its support $S_i$ is incremented by 1 (see (21)):

46 $$\begin{aligned} \varPsi _i = \frac{\sum _{i\in S_i} \psi _i}{S_i}. \end{aligned}$$

where $v_1,\ldots ,v_L$ represent the singleton consequents responsible for the (certainty of the) classification response of the corresponding rules: in our case of binary classification problems, these values are within [0, 1], where a value close to 0 represents a high certainty in Class 0 (and a low one in Class 1), and vice versa for a value close to 1. Their update based on stream samples considering uncertainty in the $\{0,1\}$-class labels provided by the user is presented in the next section. Our rules can represent certainty degrees in the rule outputs based on uncertainty provided by the user. This achieves a higher level of interpretability given the user input on the problem. Rounding the consequent values to the nearest integer provides crisp $\{0,1\}$ outputs.

The values of *g*, *u*, and $\beta$ in the AND/OR connections of the rule antecedents define how the rules can finally be read. Further details on the combinations of the values and the corresponding neurons can be found in de Campos Souza and Lughofer (2022a).

### Third layer

Finally, the output layer consists of one neuron (which can be considered as a singleton) whose activation function is given by:

47 $$\begin{aligned} y= \varOmega \left( \sum _{j=0}^{L} f_{\Gamma }(z_j,v_j)\right) \ and \ \varOmega =\left\{ \begin{array}{l}\begin{array}{cc}1,\;if\;\sum _{j=0}^{L} f_{\Gamma }(z_j,v_j)\;>0\end{array}\\ \begin{array}{cc}-1,\;if\;\sum _{j=0}^{L} f_{\Gamma }(z_j,v_j)\,<0\end{array}\end{array}\right. \end{aligned}$$

where $z_0$ = 1, $v_0$ is the bias, and $z_j$ and $v_j$, $k = 1, \ldots , L$, are the output and corresponding weight of each fuzzy neuron of the second layer, respectively, and $f_{\Gamma }$ represents the neuron activation function.

The output neural network weights are obtained by the recursive least squares (RLS) approach as described below, which is extended with the integration of the label’s uncertainty level.

### Training method

In this sub-section, the definition of the consequents of the fuzzy rules (the *v*’s), which also represent the weights that connect the second layer of the model to the neural aggregation network, is obtained in two stages, using the concepts of extreme learning machine (ELM) in the offline phase and a modified recursive least squares in the evolving stage. The latter was modified in a way such that the calculation of $\mathbf {v}$’ is also influenced by the uncertainty in user-feedback labels.

In the offline training stage, weights are determined following the extreme learning machine approach (Huang et al. 2006b):

48 $$\begin{aligned} \mathbf {v}= \mathbf {Z}_{rul}^{+} y \end{aligned}$$

where $\mathbf {Z}_{rul}^{+}$ is the Moore-Penrose pseudo-inverse (Albert 1972) of the activation matrix $\mathbf {Z}_{rul}$ ($(\mathbf {Z}_{rul}^T\mathbf {Z}_{rul})^{-1}$), which contains the activations of all neurons. This result is the least squares solution for the weights of the output layer. The $\mathbf {v}$’s values are updated on-line by using recursive least-squares (Huang et al. 2006a). This approach can be used to update the weights of the output layer as follows (Ljung 1999) (here from sample (time point) $t-1$ to sample *t*):

49 $$\begin{aligned} \eta = {} {\mathbf {z}}^{t}{Q}^{t-1}\left( \rho +({\mathbf {z}}^{t})^{T}{Q}^{t-1}{\mathbf {z}}^{t}\right) ^{-1} \end{aligned}$$

50 $$\begin{aligned} {Q}^{t} = {} (\mathrm{I}_{L^{t}}{-}\eta ^{T}{\mathbf {z}}^{t})\rho ^{-1}Q^{t-1} \end{aligned}$$

51 $$\begin{aligned} \mathbf {v}^{t}= & {} \mathbf {v}^{t-1}+\eta ^{T}(y^{t}{-}{\mathbf {z}}^{t}\mathbf {v}^{t-1}) \end{aligned}$$

where ${\mathbf {z}}$ denotes the activation levels of all neurons in the current stream sample, $\eta$ is the current kalman gain (row) vector, ${I}_{L_{s}^{t}}$ is an identity matrix based on the number of neurons (*L*) in the second layer, $L_{s}^{t}\times L_{s}^{t}$, *Q* denotes the inverse Hessian matrix $(\mathbf {Z}_{rul}^T\mathbf {Z}_{rul})^{-1}$ and is set initially to $\omega \mathrm{I}_{L_{s}^{t}}$, where $\omega$=1000 as in Rosa et al. (2014), and is then continuously updated directly by Eq. (50) without requiring any re-inversion of matrices; $\rho$ is a forgetting factor and typically set to values between 0.9 and 1.

An intuitive way of integrating the uncertainty information provided by the user, would be to use a fuzzy value that reflects the uncertainty degree and falls between the two crisp values of 0 and 1 for the two classes; for instance, if the user provided an uncertainty level of 0.2 together with class label 1, then *y* could be set to 0.8 (rather than to 1, which would be the classical case without uncertainty information), and then Eq. (51) would be used for updating. This would induce a movement of the *v*’s away from 1 towards 0.8. However, the problem with such an integration is that it assumes reciprocal uncertainty for the two classes: this means that an uncertainty of 0.2 in Class label 1 automatically assumes an uncertainty of 0.8 in Class label 0. This may not necessarily be the case, as samples may be labeled with high uncertainty in both classes (e.g., 0.8 and 0.9), and the user chooses the less uncertain class as final label. In such a case, a full update would be conducted with a value close to 0, which would then incorrectly reflect a low uncertainty in class label 0. Therefore, we integrate the uncertainty in the class label of the current sample $\psi _t$ in (51) resulting in the new approach termed as *RLS-U (recursive least squares with uncertainty integration)* as follows:

52 $$\begin{aligned} \mathbf {v}^{t}=\mathbf {v}^{t-1}+\eta ^{T}(y^{t}{-}{\mathbf {z}}^{t}\mathbf {v}^{t-1})(1-\psi _t) \end{aligned}$$

Uncertainty is thus integrated by a multiplication of $(1-\psi _t)$ with the current prediction error (multiplied with Kalman gain). The effort for updating the current consequent vector *v* (relative to the error) is low when the uncertainty is high (independent of the label assigned) and high (or full) when the uncertainty is low (or zero as in the classical case). Samples with labels indicating a high uncertainty therefore have a little influence on the model’s update, which is desired. The training algorithm for our ENFC-U approach can be summarized in Algorithm 1.
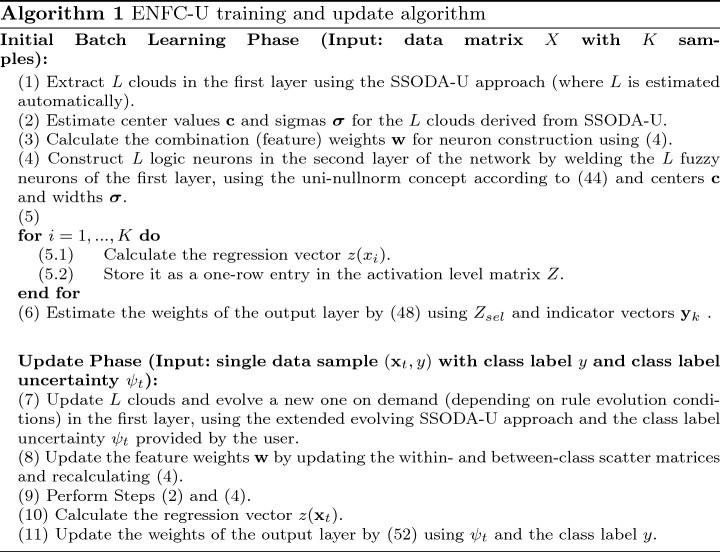


## Results and discussion

### Preliminary test definitions and evaluation criteria

In the experiments, various uncertainty levels were introduced in order to simulate users’ certainty degrees on class labels and their experience levels. It is known that uncertainty can drastically affect the accuracy of data-driven and machine learning models (Lewis and Catlett 1994). Therefore, when introducing uncertainty in this context, the final impact on model accuracy should be ideally lower than the uncertainty level introduced by the user. Thus, we evaluated our approach by comparing the classification accuracy on data containing no uncertainty with the accuracy achieved on data including an associated uncertainty level of X%. Hence, when an average data uncertainty of around X% is introduced, the accuracy of the model obtained from the learning algorithm should decrease by less than X%.

In this work, we chose classification accuracy as stream evaluation criterion. This form of verification compares the result predicted by the model with that obtained in the experiments and can be expressed by:

53 $$\begin{aligned} Accuracy=\frac{TP+TN}{TP+FN+TN+FP} \end{aligned}$$

where $TP =$ the number of true positives, $TN =$ the number of true negatives, $FN =$ the number of false negatives and $FP=$ the number of false positives. In the tests described below, accuracy was combined with a trend line approach to adapt it to the stream mining case. In this context, the one-step-ahead prediction accuracy is updated cumulatively by:

54 $$\begin{aligned} Accuracy(K+1) = \frac{Accuracy(K) * K + I_{\hat{y}=y}}{K+1}, \end{aligned}$$

where *I* denotes the indicator function, which equals 1 when the prediction is correct, (i.e., $\hat{y}=y$) and 0 otherwise ($Accuracy(0) = 0$); subsequently, the model is updated. This results in an interleaved test-and-then-train scenario, which is widely used in the stream mining community, see Bifet et al. (2010). Note that for *y* we always use the given (ground truth) labels in the stream, and that the accuracy at each sample *K* can be stored in a vector, to create an accuracy trend line (as shown further below for various data streams).

All feature samples in the experiments were normalized such that they had a mean equal to zero and a standard deviation equal to one. The uncertainty provided by the user was sampled randomly. Uncertainty intervals were defined to identify model efficiency for various levels of sample uncertainty. Thus, we ran tests where the average uncertainty was zero, simulating the data in its original form, and tests with uncertainty in the ranges 0–10%, 10–20%, and 20–30% to simulate low, average and high sample uncertainty, respectively, in order to assess the (general) robustness of our method with respect to these uncertainty levels (i.e., to check how much the accuracy trend lines deteriorate in these cases).. Ten tests were performed on different shuffles of the data in each experiment in order to better visualize significant differences along the accumulated accuracy trends (as all were then surrounded by a one-sigma area among the ten runs). Furthermore, we performed statistical preference analysis tests based on the accuracy trends, in order to check for statistically significant differences in the trend lines.

Furthermore, in order to be able to check the performance of our extensions to SODA (SSODA-U) and RLS (RLS-U), where class label uncertainty is explicitly integrated (as discussed in the methodological section above), we compared them with the original SODA and RLS variants (which do not consider uncertainty levels) on modified data streams from our application scenarios (see below), where we simulate X% uncertainty by changing the original (ground truth) class labels in X% of the samples and used the X% uncertainty level as $\psi$-values in our extensions (the original variants cannot use these $\psi$-values). This mimicked the case in which less experienced users label the data.

### Cyber attack’ identification tests

Several types of hacker actions can interfere with computers’ proper functioning and systems that depend on the internet. Simulated attack data is commonly applied to hybrid models (Souza et al. 2020; Campos Souzaet al. 2020; Batista et al. 2019). Such data sets can help to detect anomalous behavior in the internet. In this experiment, two data sets were used, one simulating SQL injection and the other represents malware attacks. Table 1 shows the basic properties of the data used in the tests.

**Table 1:** Cyber-attack datasets

Dataset	Acronym	Source	Attacks	Normal commands
SQLDataset.data	SQLD	Demertzis and Iliadis (2015)	12.881	1.003
VirusDataset.data	MALD	Perdisci et al. (2008)	669	2.598

**Fig. 4:**
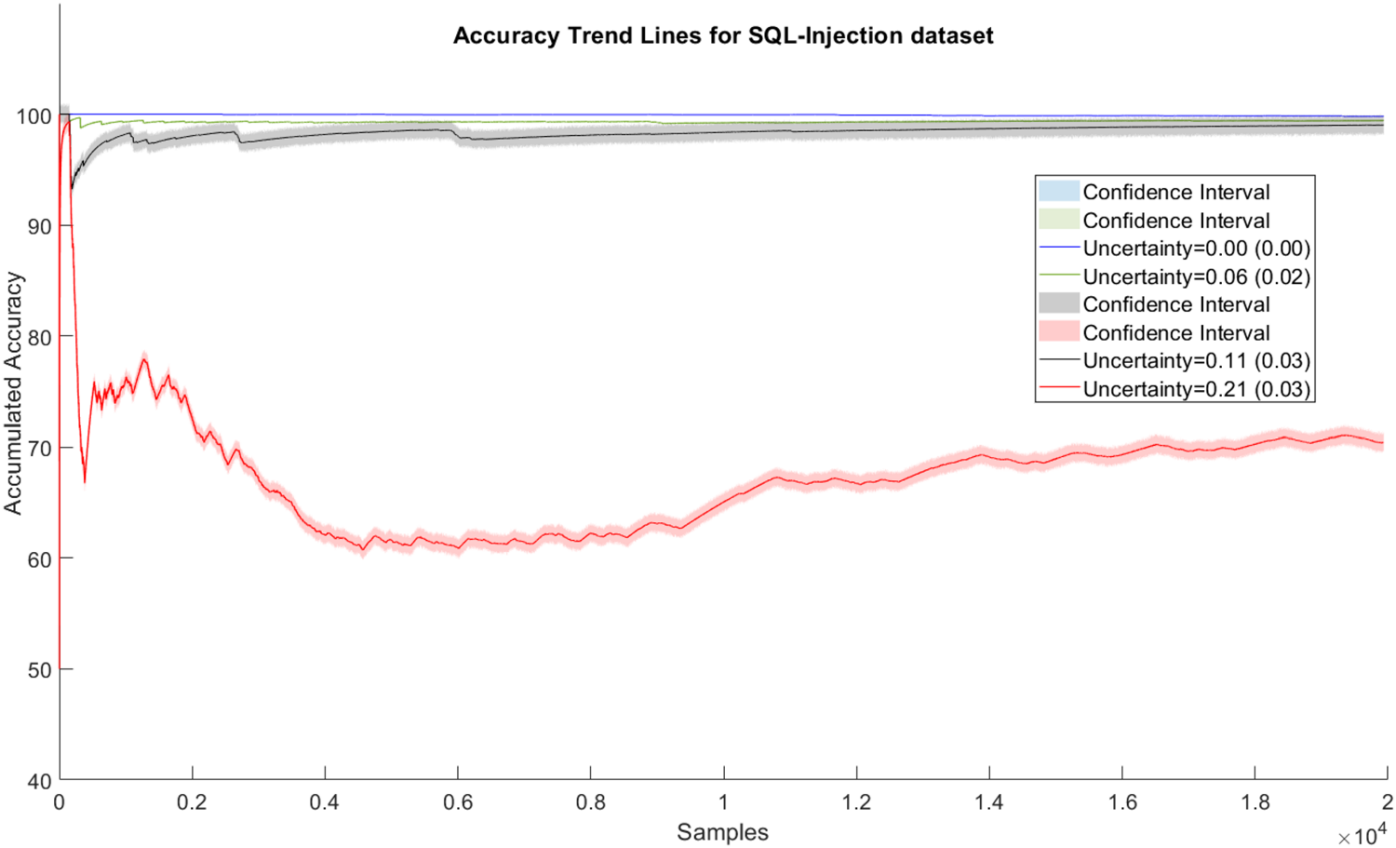
Accuracy trend lines of SQL injection—SQLD data set

**Fig. 5:**
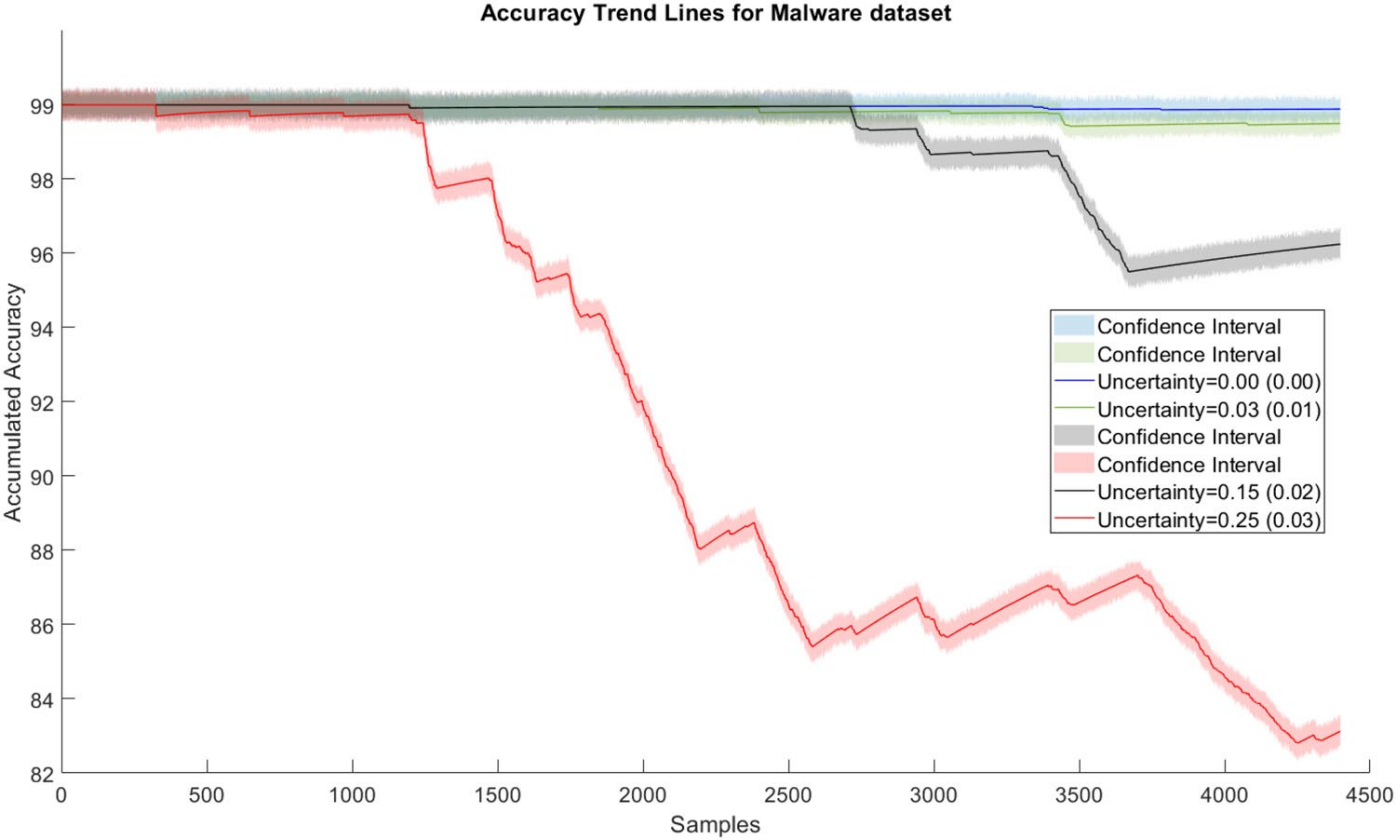
Accuracy trend lines of Malware—MALD data set

The accuracy trend lines as calculated by (54) are shown in Figs. 4 and 5 for SQL injection and malware data streams, respectively.

The results for SQL injection invasions demonstrate our approach’s viability because, despite using data with considerable degrees of uncertainty (up to 11%), the performance was minimally impacted. Also, the loss in accuracy stands out when data uncertainty becomes significantly higher (around 20%). The results for high uncertainty demonstrate the training method’s difficulty in identifying new samples. The model’s ability to maintain accuracy when identifying cyber attacks (99.6% versus 98.9%) for data uncertainties (0% versus 6%) was outstanding. In the case of the malware data stream, the difference in the accuracy between models using data with and without uncertainty was greater. Especially, in the case when the uncertainty became more than 20%, a significant down-trend could be observed (red line in the figures). This somehow provided us achievable bounds of our approach. For the lower uncertainty level cases, the accuracy loss was much lower than the uncertainty increase, e.g., (99.8% with 0% uncertainty versus 99.2 with 5% uncertainty, thus only 0.6% lower, although the uncertainty level was 5% higher). This proves that the model can well cope with data uncertainty up to some level in a robust manner. Or in other words, up to a certain level our approach is robust in incorporating user input on class label uncertainty into model training.

### Auction fraud identification

Using a well-known data set, we present the initial rules extracted from the data sets and how (much) these change due to further evolution on a separate online set.

A commonly known problem in online commerce is auction fraud, particularly in shill bidding (SB) practices, where auxiliary accounts to increase the bids on products in active auctions. Analysis of this type of behavior is complex as it involves various uncertainties, which makes it a perfect problem for testing our approach. To detect these practices, Alzahrani and Sadaoui compiled a data set of harmful practices in auctions with 6321 samples including standard and fraudulent bids Alzahrani and Sadaoui (2018). The original data set has 12 input features, but for our purposes the dimensions related to personal identification (Record ID, Auction ID and Bidder ID), which are identification values and not values collected in the experiments, were irrelevant and thus removed. The remaining nine dimensions are listed in Table 2.

**Table 2:** Input features of the auction problem Alzahrani and Sadaoui (2018)

Feature	Description
Bidder tendency	An SB participates exclusively in auctions of few sellers rather than a diversified lot to help in this cheating
Bidding ratio	An SB participates more frequently to raise the auction price and attract higher bids from legal participants
Successive outbidding	An SB always outbids himself even though he is the current winner, slowly increasing the price with small values
Last bidding	An SB becomes inactive when the auction is near to 90% of its duration to avoid winning
Auction bids	auctions with dubious activities tend to have a much higher number of bids than the average
Auction starting price	An SB usually offers a modest starting price to invite legitimate bidders into the auction
Early bidding	An SB tends to bid in less than 25% of the auction duration to get the attention of auction users
Winning ratio	An SB competes in many auctions but rarely wins any
Auction duration	How long an auction remained
Class	0 for normal behaviour bidding; 1 otherwise

**Fig. 6:**
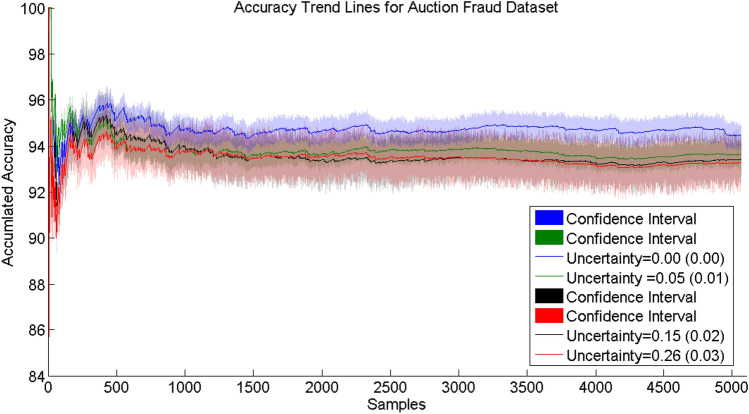
Accuracy trend lines for auction fraud data sets

The tests to identify fraud in auctions were carried out comparing models based on data with 0% and an average of 5% uncertainty. The results are shown in Fig. 6.

The results demonstrate that our approach of including expert input on uncertainty in the data works well in the context of detecting auction fraud. The results are similar to those of approaches that use SVM (support vector machine) techniques to solve this problem (Anowar et al. 2018). To prove this statement, a *t*-test was performed where the reference value (0.940) was obtained in Anowar et al. (2018). The results are listed in Table 3,^1^

**Table 3:** *t*-Test for auction fraud data set test results with a $95\%$ confidence level

Model	*t*-value	*p*-value
Uncertainty (0.00)	1.42	0.82*
Uncertainty (0.05)	0.83	0.54*
Uncertainty (0.15)	0.21	0.06*
Uncertainty (0.26)	0.07	0.05*

Even with an average of 5% uncertainty, our results were on average only less than 1% less accurate than those of the model that was evolved on the data with 0% uncertainty. Interestingly, for this data stream the accuracy trend line decreased only minimally when increasing the average uncertainty level further to 15% and even to 26%, which emphasizes the robustness of our approach. Additionally, we analyzed the fuzzy rules extracted in the context of fraud identification. The cloud filter for samples with a high degree of uncertainty worked well, as both models ultimately created the same number of fuzzy rules, thus knowledge extraction was not affected by uncertainty. The interpretability of the rules is a significant factor in understanding how the model changes depending on the level of uncertainty and how it can generate knowledge. Tables 4 and 5 present the degree of change in the model without uncertainty and with uncertainty contained in the data, respectively, based on the structural changes tracking approach as proposed in de Campos Souza and Lughofer (2021).

**Table 4:** Interpretability with respect to (the degree of) changes in fuzzy neurons during the evolution phase without uncertainty

Rule 1 changed in 3 membership functions and by a degree of 0.01 with no consequent change
Rule 2 did not change with no consequent change
Rule 3 changed in 5 membership functions and by a degree of 0.06 with no consequent change

**Table 5:** Interpretability with respect to (the degree of) changes in fuzzy neurons during the evolution phase with uncertainty

Rule 1 changed in 2 membership functions and by a degree of 0.03 with no consequent change
Rule 2 did not change with no consequent change
Rule 3 changed in 5 membership functions and by a degree of 0.08 with no consequent change

There are differences between the connectives and the membership functions of the antecedents under the fuzzy rules generated. Thus, knowledge about auction fraud can be better interpreted, the impact of uncertainty on the fuzzy rules generated can be explored. The fuzzy rules generated without data uncertainty are presented below:

1. If Bidder Tendency is Small with impact 0.02 or Bidding Ratio is Small with impact 0.11 or Successive Outbidding is Small with impact 1.00 or Last Bidding is Small with impact 0.00 or Auction Bids is Small with impact 0.00 or Auction Starting Price is Small with impact 0.00 or Early Bidding is Small with impact 0.00 or Winning Ratio is Small with impact 0.04 or Auction Duration is Small with impact 0.00 then Auction Fraud is Yes with certainty 0.36 and average (rule) uncertainty of 0%.
2. If Bidder Tendency is Medium with impact 0.02 and Bidding Ratio is Medium with impact 0.11 and Successive Outbidding is Medium with impact 1.00 and Last Bidding is High with impact 0.00 and Auction Bids is High with impact 0.00 and Auction Starting Price is Medium with impact 0.00 and Early Bidding is High with impact 0.00 and Winning Ratio is Medium with impact 0.04 and Auction Duration is Medium with impact 0.00 then Auction Fraud is No with certainty 0.01 and average (rule) uncertainty of 0%.
3. If Bidder Tendency is Medium with impact 0.02 or Bidding Ratio is High with impact 0.11 or Successive Outbidding is Medium with impact 1.00 or Last Bidding is Medium with impact 0.00 or Auction Bids is Medium with impact 0.00 or Auction Starting Price is High with impact 0.00 or Early Bidding is Medium with impact 0.00 or Winning Ratio is High with impact 0.04 or Auction Duration is High with impact 0.00 then Auction Fraud is Yes with certainty 1.00 and average (rule) uncertainty of 0%.

The changes generated in the fuzzy rules when uncertainty was included demonstrate that our approach can update the Gaussian centers in the first layer, which brings new knowledge about the problem. The fuzzy rules generated by simulating data that incorporates user input are:

1. If Bidder Tendency is Small with impact 0.02 or Bidding Ratio is Small with impact 0.11 or Successive Outbidding is Small with impact 1.00 or Last Bidding is Small with impact 0.00 or Auction Bids is Small with impact 0.00 or Auction Starting Price is Small with impact 0.00 or Early Bidding is Small with impact 0.00 or Winning Ratio is Small with impact 0.04 or Auction Duration is Small with impact 0.00 then Auction Fraud is Yes with certainty 0.21 and data average (rule) uncertainty of 5%.
2. If Bidder Tendency is Medium with impact 0.02 or Bidding Ratio is Medium with impact 0.11 or Successive Outbidding is Medium with impact 1.00 or Last Bidding is High with impact 0.00 or Auction Bids is High with impact 0.00 or Auction Starting Price is Medium with impact 0.00 or Early Bidding is High with impact 0.00 or Winning Ratio is Medium with impact 0.04 or Auction Duration is Medium with impact 0.00 then Auction Fraud is No with certainty 0.00 and data average (rule) uncertainty of 5%.
3. If Bidder Tendency is Medium with impact 0.02 and Bidding Ratio is High with impact 0.11 and Successive Outbidding is Medium with impact 1.00 and Last Bidding is Medium with impact 0.00 and Auction Bids is Medium with impact 0.00 and Auction Starting Price is High with impact 0.00 and Early Bidding is Medium with impact 0.00 and Winning Ratio is High with impact 0.04 and Auction Duration is High with impact 0.00 then Auction Fraud is Yes with certainty 1.00 and data average (rule) uncertainty of 5%.

Another factor to be highlighted is that the model was able to identify the most relevant feature in the auction fraud mode, which is the practice of successive outbidding. Other features were rated as irrelevant (value 0) and some as being of low relevance. Thus, fuzzy rules can be seen as more straightforward and easy to understand by humans. An example of a fuzzy rule customized by taking into account the feature weights (features with weights of 0 can be omitted) is presented below:

If Bidder Tendency is Medium with impact 0.02 and Bidding Ratio is High with impact 0.11 and Successive Outbidding is Medium with impact 1.00 and Winning Ratio is High with impact 0.04 then Auction Fraud is Yes with certainty 1.00 and average (rule) uncertainty of 5%

If Bidder Tendency is Medium with impact 0.02 or Bidding Ratio is Medium with impact 0.11 or Successive Outbidding is Medium with impact 1.00 or Winning Ratio is Medium with impact 0.04 or then Auction Fraud is No with certainty 0.00 and average (rule) uncertainty of 5%.

This reduces the antecedent parts of the fuzzy rules from 9 down to 4, resulting in a much more readable rule base. Due to a comparison of the fuzzy rules, it is possible to verify the elements that favor one behavior over another. For the identification of fraud, the bidding ratio and winning ratio dimensions stand out. When they are present at high frequency, there is a greater likelihood of fraud. The first represents fraud with high certainty, and the second represents normal behavior in the auction with high likelihood.

#### Comparison tests with orginal model updates

In order to be able to check the performance of our extensions to SODA (SSODA-U) and RLS (RLS-U), which explicitly integrate class label uncertainty, we compared them with the original variants SODA and RLS (which do not consider uncertainty) on modified data streams from our application scenarios (see below), where we simulated X% uncertainty by changing the original (ground truth) class labels in X% of the samples and used the X% uncertainty level as $\psi$-values in our extensions (the original variants cannot use these $\psi$-values). This simulated the case in which an unexperienced user labels the data. We expected our new approach EFNC-U which uses SSODA-U and RLS-U, to be superior to the traditional SODA+RLS approach. Almost 10%, 20% and 30% of the sample labels were changed randomly. Therefore, we intend to compare whether the propositions made in this paper (an improvement on a previously published model) when evaluating the uncertainty of labels improves the model’s efficiency in identifying the change of labels.

**Fig. 7:**
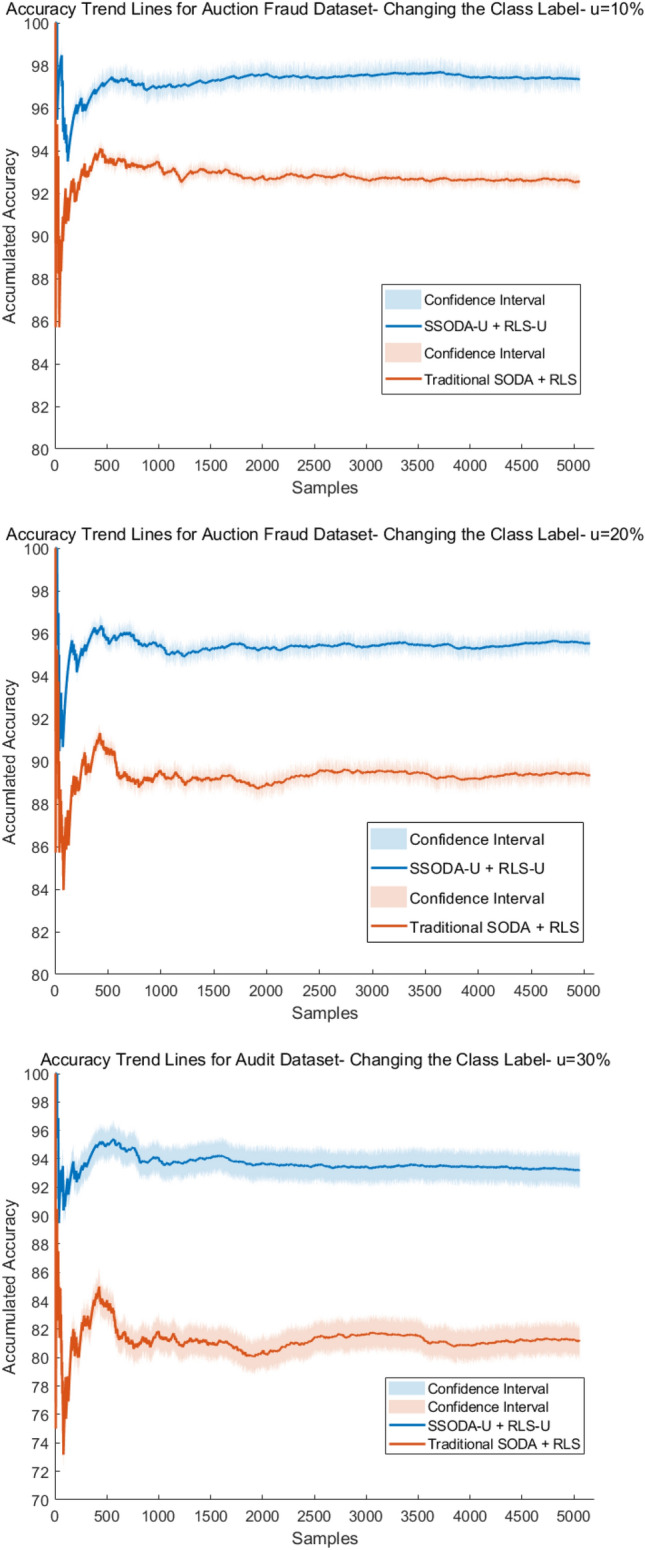
Accuracy trend lines of auction fraud data set with X% changed labels

**Fig. 8:**
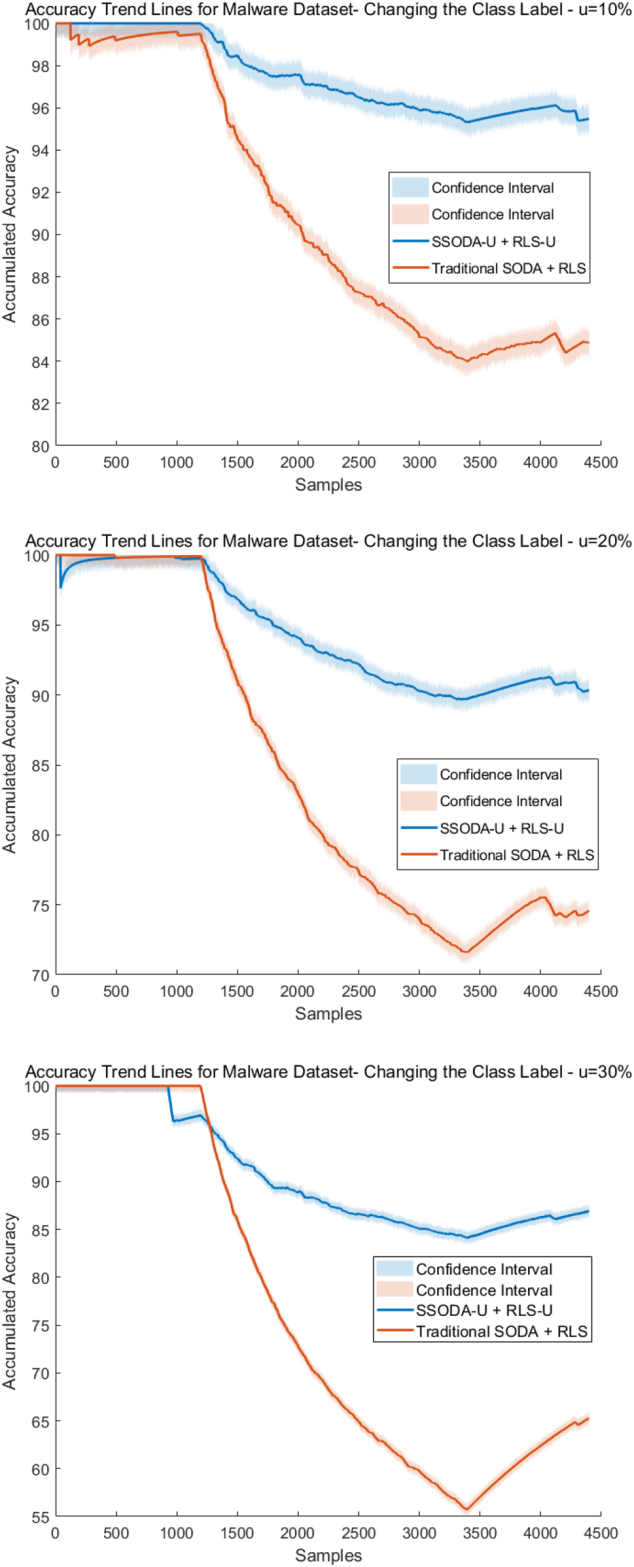
Accuracy trend lines of malware data set with X% changed labels

**Fig. 9:**
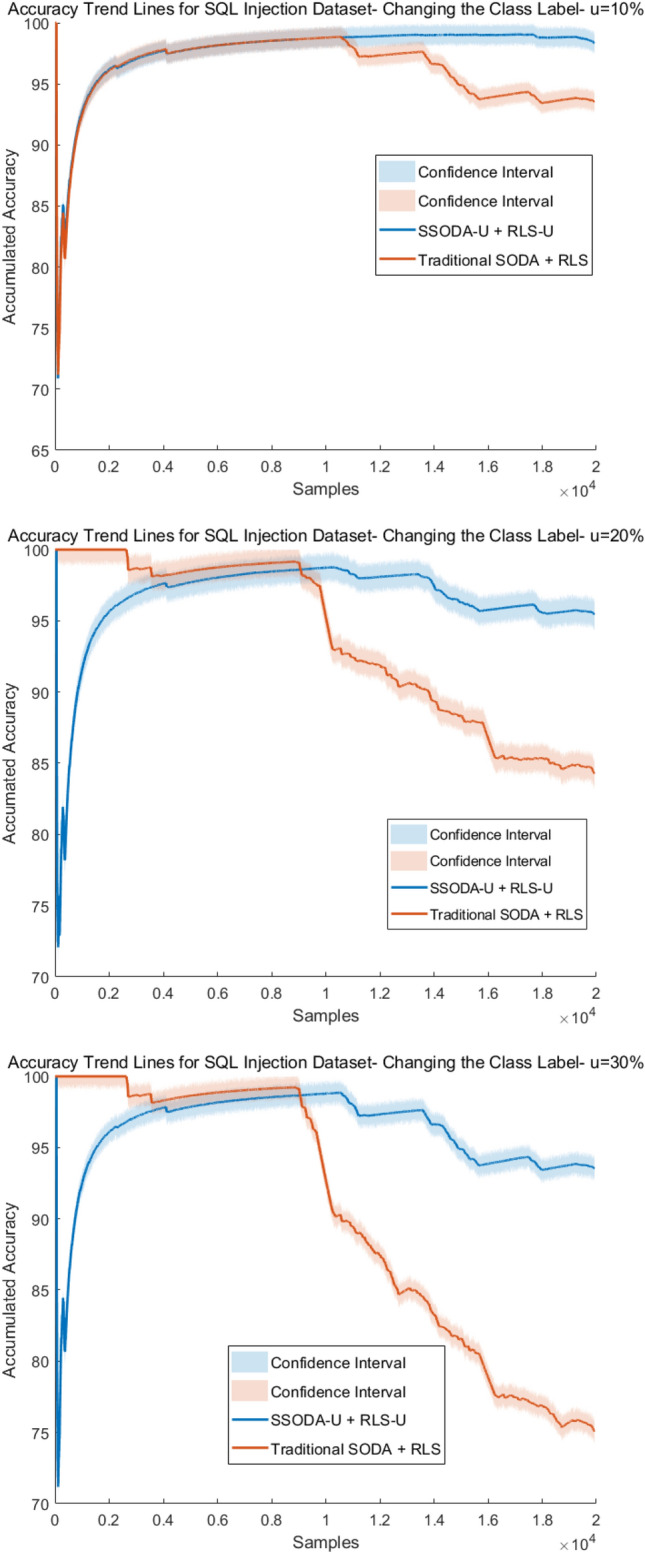
Accuracy trend lines of SQL injection data set with X% changed labels

Figures 7, 8 and 9 illustrate the results.

It can be seen that, especially in the second phase of the streams, the accuracy trend lines differ significantly as they are affected by uncertainty, and that the behavior differs between the two approaches. For instance, for the auction fraud data set and 10% uncertainty, about 4% higher accuracies were achieved with our approach than with the original SODA+RLS in the stream modeling process (compare the blue with the red line), while the difference is also noted to increase between the proposal of this paper and the traditional model (approximately 6% difference with 20% uncertainty and 10% for 30% uncertainty). In the case of the malware data set, the differences between the approaches were more significant and increasing (approximately 10%, 15% and 22% difference in accumulated accuracy for 10, 20 and 30% uncertainty respectively); this again shows high robustness with respect to uncertainty, and in all cases our approach outperformed traditional SODA+RLS. In the case of the SQL injection data set, the drop in the accuracy, concerning increasing uncertainty levels, during the test behavior is also highlighted as the uncertainty about the labels increases for traditional SODA+RLS.

Overall, our model achieved better accuracy results (in most cases and was at least on a par with) the traditional SODA model in the rest of the cases. This means that explicit integration of uncertainty levels in the update of the clouds and the consequent weight vectors resulted in a robust learning behavior. Such uncertainty levels can be used, for instance, in ML interaction/annotation systems when users with less experience than a super-user (who knows the ground truth) (start to) label the data.

The statistical analysis of variance (ANOVA) (St and Wold 1989) test was conducted to verify if the approaches proposed in these tests achieved the same performance when classifying each of the data sets with different levels of uncertainty. After its execution, it was found that the models did not have similar performances in any of the test groups (as achieving a *p*-value of 0.021 for the auction fraud test, a *p*-value of 0.007 for the malware test, and a *p*-value of 0.029 for the SQL injection test).

To verify the assumptions of the ANOVA results, three tests were used to confirm it: normality, homoscedasticity, and independence of data (Montgomery 2017). We confirmed the data normality conditions using the Shapiro–Wilk test. The homoscedasticity that verifies the equality of variances of the residues was validated using the Fligner–Killeen test, resulting in similar variances in all the classification performances. Finally, the Durbin–Watson test confirmed that the data under analysis is independent. All the results are listed in Table 6.

**Table 6:** Assumptions of the ANOVA results: *p*-values

Test	Normality	Homoscedasticity	Independence
Auction fraud	0.0910	0.7114	0.4217
Malware	0.0598	0.5165	0.3130
Sql Injection	0.0741	0.4320	0.1220

Thus, we conclude with 95% confidence that the models have different accuracy performances in each of the tests performed.

Once we verified the specific performances in the models, we conducted a post-hoc Tukey test for multiple comparisons, one-to-one, among all the models involved in the benchmark. The results are listed in Tables 7, 8 and 9 (* means that the results are statistically equivalent with respect to a confidence interval of 95%). Based on these results, the conclusion is, with 95% confidence, that the model proposed in this paper has a better behavior to understand the changes in the labels, and it can evolve with a higher performance than the related SoA method (conventional SODA+RLS).

**Table 7:** Post-hoc Tukey test results for auction fraud data set with a $95\%$ confidence level

Model–Model	Difference	Lower	Upper	*p*-value
**SSODA-U+RLS-U—SODA+RLS (10%)**	3.12	$-0.77$	2.12	0.03
**SSODA-U+RLS-U—SODA+RLS (20%)**	4.87	$-1.18$	3.49	0.02
**SSODA-U+RLS-U—-SODA+RLS (30%)**	5.39	$-2.06$	6.82	0.01

**Table 8:** Post-hoc Tukey test results for malware data set (MALD) with a $95\%$ confidence level

Model–Model	Difference	Lower	Upper	*p*-value
**SSODA-U+RLS-U—SODA+RLS (10%)**	7.77	$-0.81$	4.82	0.02
**SSODA-U+RLS-U—SODA+RLS (20%)**	9.22	$-1.19$	6.31	0.01
**SSODA-U+RLS-U—SODA+RLS (30%)**	9.81	$-1.82$	8.20	0.00

**Table 9:** Post-hoc Tukey test results for SQL injection dataset (SQLD) with a $95\%$ confidence level

Model–Model	Difference	Lower	Upper	*p*-value
**SSODA-U+RLS-U—SODA+RLS (10%)**	1.12	$-0.19$	1.98	0.05*
**SSODA-U+RLS-U—SODA+RLS (20%)**	5.21	$-0.45$	7.74	0.03
**SSODA-U+RLS-U—SODA+RLS (30%)**	9.39	$-1.74$	9.74	0.01

## Conclusion

ENFC-U, the evolving fuzzy neural classifier (ENFC) presented in this paper, incorporates user feedback on class label uncertainty in model training, adaptation and evolution. The results from cyber-attack tests and fraud assessment in auctions confirm the idea that it is possible to incorporate this uncertainty, while maintaining model accuracy of the problem analyzed. Furthermore, the model tests showed that there is a limit for the uncertainty level regarding robustness of the models predictions (measured in terms of accuracy trend lines). When the average uncertainty associated with the data was higher than 20%, the model lost its ability to correctly identify data labels, as its predictive performance was significantly deteriorated. Therefore, in addition to being a relevant factor for training the model, it also becomes its weakness because the levels of accuracy fall to levels greater than the uncertainty associated with the samples. The results are, however, robust when the uncertainty in the provided labels is less than 20%, as then the effect on the accuracy trend lines was negligible. The fuzzy rules generated by the evolving fuzzy neural network model contributed reliably to the identification of the classification problems, thus offering some sort of useful knowledge extraction. The generated fuzzy rules showed good interpretability, as they could be greatly reduced by its length due to the integration of feature weights indicating the impact of features on the classification problem. This factor allows a synergy between the model proposed in this paper and the user experience in adequately evaluating the samples involved in a problem evaluation process. By associating uncertainty with fuzzy rules consequents, experts can gain a basis for how strong a rule is favoring a certain class among the others in that particular region it (geometrically) represents. Furthermore, the uncertainty levels in the class labels did hardly affect the knowledge finally extracted, which indicates another form of robustness of our method.

Future work will be related to the incorporation of user uncertainty into other procedures, for instance, by setting weights for fuzzy neurons or other training forms. Further, it would be interesting to incorporate another type of user feedback into the model’s update procedure, that is not related to the data, but, for example, to the output of the model or to its input structure (typically in form of expert rules). Another possible extension to this work would be to evaluate the data uncertainty in the definition of synaptic weights.

## Data Availability

This study was a re-analysis of existing data. The Auction fraud identification dataset analysed is available in the UCI Machine Learning Repository: https://archive.ics.uci.edu/ml/datasets/Shill+Bidding+Dataset. The Virus Data set is available in the Researchgate: https://www.researchgate.net/publication/328391682_virus_full. In the case of data sets referring to SQL Injection, it was obtained upon request from the researcher Konstantinos Demertzis, author of the paper that deals with this data set (Demertzis and Iliadis 2015).

## Footnotes

1 (* means that the results are statistically equivalent with respect to a confidence interval of 95%).
